# Prescription opioids and economic hardship in France

**DOI:** 10.1007/s10198-022-01557-4

**Published:** 2023-01-30

**Authors:** Ilaria Natali, Mathias Dewatripont, Victor Ginsburgh, Michel Goldman, Patrick Legros

**Affiliations:** 1 Université Libre de Bruxelles, Avenue F. D. Roosevelt, 50, 1050 Brussels, Belgium; 2 European Center for Advanced Research in Economics and Statistics (ECARES), Université Libre de Bruxelles, Brussels, Belgium; 3 Institute for Interdisciplinary Innovation in Healthcare (I3h), Université Libre de Bruxelles, Brussels, Belgium; 4 Toulouse School of Economics (TSE), Université Toulouse 1 Capitole, Toulouse, France

**Keywords:** Prescription opioids, Socioeconomic conditions, France, Opioid crisis, I11, I15, I18

## Abstract

This paper studies how opioid analgesic sales are empirically related to socioeconomic disparities in France, with a focus on poverty. This analysis is made possible using the *OpenHealth* database, which provides retail sales data for opioid analgesics available on the French market. We exploit firm-level data for each of the 94 departments in Metropolitan France between 2008 and 2017. We show that increases in the poverty rate are associated with increases in sales: a one percentage point increase in poverty is associated with approximately a 5% increase in mild opioid sales. Our analysis further shows that opioid sales are positively related to the share of middle-aged people and individuals with basic education only, while they are negatively related to population density. The granularity and longitudinal nature of these data allow us to control for a large pool of potential confounding factors. Our results suggest that additional interventions should be more intensively addressed toward the most deprived areas. We conclude that a combination of policies aimed at improving economic prospects and strictly monitoring access to opioid medications would be beneficial for reducing opioid-related harm.

## Introduction

During the last couple of decades, the use (and abuse) of opioid pain medications has remarkably increased in many countries worldwide, thus raising serious concerns due to the addictive nature of these substances. As we will show, in France, opioid analgesic retail sales increased for both mild and strong opioids between 2008 and 2017, opioid-related hospitalizations rose by 167% over the period 2000–2017, and opioid-related deaths by 192% over the period 2000–2016 (see Figs. 2 and 4 in Appendix 2). The number of intoxications directly linked to opioid analgesics doubled between 2005 and 2016 [2], while the popular newspaper Le Monde reports that opioid addiction is now the first cause of death by overdose in France.^1^ These increasing trends have recently induced French health authorities to implement policies aimed at restricting the use of opioid pain relievers. Identifying those risk factors favoring the abuse of opioid painkillers would greatly support decision-makers in the elaboration of suitable policy interventions and in the evaluation of their impact on the population of patients. This paper contributes in this direction, by studying how opioid analgesic sales are empirically related to socioeconomic disparities in France, with a focus on the role played by economic hardship.

Most of the health economics literature studying the adverse consequences of prescription opioid abuse has been developed in the US, where this phenomenon has caused the worst drug epidemic in the country’s history, known as the ‘opioid crisis’. Empirical evidence in this literature suggests that the intensity of the epidemic tends to be highly heterogeneous across regions, and there have been simultaneous increases in opioid use and measures of economic hardship. This has led several researchers to postulate the existence of a relationship between opioid consumption and economic conditions. Understanding this relationship is crucial for guiding policy-makers on how to address pharmacovigilance efforts and services for addiction treatment, support studies on problematic use, and help protect the most vulnerable people. This becomes now even more important, since the outbreak of the COVID-19 pandemic has strongly hit the most vulnerable strata of society and, hence, it is likely to exacerbate the effects of opioid abuse and misuse [34].^2^ This type of studies may also help predict the impact of supply-side health policies applied at the national level: any intervention aimed, for instance, at limiting access to these narcotics is likely to have a different impact across different geographical areas, depending on their socioeconomic situation.^3^

Although research efforts in this direction have been made in the US, studies on this topic in Europe are scant, while some authors (e.g., Maclean et al., [42]) highlight the importance of studying opioid misuse in countries other than the US. Moreover, in the US opioid literature, the role played by the economic climate is still debated. In this paper, we contribute by documenting the magnitude of opioid analgesic use in France and investigating the role of demand-side (economic) factors in this country. The French health, economic and social systems, as well as laws regulating access to narcotic medications, are profoundly different from those in the US. These institutional differences may affect the patterns of consumption, prescription by doctors, and the way opioid use relates to indicators of economic opportunity. We exploit the *OpenHealth*^4^ database that enables access to firm-level retail sale data for opioid analgesics available on the French market between 2008 and 2017 and the 94 departments in Metropolitan France.^5^ We investigate sales trends at the national level for each opioid active ingredient as well as the relationship between the use of opioid painkillers and local economic conditions, controlling for other socio-demographic indicators. While most of the literature on the relationship between economic opportunity and prescription opioid (mis)use focuses on (un)employment measures, we consider the poverty rate instead. This is because we believe that the prevalence of poverty represents a better measure of economic distress, especially in France, where public authorities provide significant financial support to unemployed individuals. As mentioned by Deaton [21], “deaths of despair [...] respond more to prolonged economic conditions than to short-term fluctuations”. Moreover, prior research typically uses aggregate measures of opioid prescriptions and/or opioid-related harm as outcome variables, which often does not allow to distinguish between licit and illicit use and/or across substances. To the best of our knowledge, we are the first to exploit firm-level local data. This is important because the longitudinal nature and granularity of data provided by *OpenHealth* allow us to control for a vast pool of potential confounding factors and, hence, to evaluate the impact of demand-side determinants (i.e., the prevalence of poverty) independently of supply-side factors (e.g., the presence of high-frequency prescribers at the local level and pharmaceutical companies’ marketing) and of policy interventions (given the centralized nature of the French health system). In addition, these data allow us to study the relationship with economic conditions for each opioid active ingredient separately, thus uncovering different patterns across substances.^6^ Finally, we test the sensitivity of our results to alternative definitions of poverty and alternative specifications.

The paper is organized as follows. In “Literature review”, we summarize the relevant literature, and in “Institutional background”, we provide a brief overview of the French health system. We describe our data and provide summary statistics in “Data and summary statistics”. We discuss consumption trends and substitution patterns among several opioid analgesics on the French market in “Nationwide consumption and substitution patterns”. “Econometric analysis” contains the econometric analysis and discusses the results. We conclude in “Conclusions and policy implications”.

## Literature review

This paper is related to, at least, three strands of the health economic literature: the literature studying geographical variations in healthcare utilization; the literature investigating the relationship between economic conditions and health; the recent, growing literature on the US opioid crisis. In the following subsections, we summarize the main findings from these studies.

### Geographical variation in healthcare utilization

This paper contributes to a line of research studying geographical variation in healthcare utilization. Several papers have explored this phenomenon in France. Indeed, despite the centralized nature of the French healthcare system and the compulsory character of the health insurance, there seem to be inequalities in access and quality of care across geographical areas in France. Close to our paper is, for example, the work by Beuscart et al. [4]. The authors investigate the incidence of potentially inappropriate medication (PIM) prescribing for the population of elderly patients in the French region Nord-Pas-de-Calais, and find that there is substantial variation in PIM across municipalities. Interestingly, PIM is more prevalent in municipalities with higher levels of unemployment and lower household income. Mercier et al. [44] evaluate, instead, variation in potentially avoidable hospitalizations at the zip code level and find that these are more common in areas characterized by lower levels of education and income. Importantly, they also find a positive and significant association between avoidable hospitalizations and deaths, thus confirming that variations in quality and provision of care have a strong impact on patients’ health status. In France, there also exists variation in admissions for ambulatory care sensitive conditions (ACSCs) across departments, as shown in Weeks et al. [67]. The prevalence of ACSCs is often used in the literature to proxy poor quality of primary care. These authors find that ACSCs are negatively correlated with income and it is more common in France than in other countries under study. Weeks at al. [66] find that considerable heterogeneity across French departments also exists for elective surgery use, and utilization rates are lower than in the US. Rococo et al. [54] focus, instead, on breast cancer surgery to evaluate the degree of accessibility to high-quality procedures and find substantial disparities across French hospitals. Lonjon et al. [40] uncover variation in spine surgery procedures implemented across surveyed surgeons, while Phelip et al. [52] unveil differences in diagnosis and management of rectal cancer across French regions. Admissions into ICU for elderly patients also greatly vary across hospitals [6]. Finally, Mousquès et al. [45] find significant variation in medical practice style even *within* a physician. They show that, in France, much of the variation in prescribing (70%) occurs *within* a physician and patients’ characteristics explain most of this variation. Unemployed individuals, for instance, receive fewer antibiotic prescriptions than active workers.

For what concerns opioid prescribing, a recent study by Silhon et al. (2020) finds that opioid pain relievers are prescribed more frequently in areas characterized by a low density of GPs.^7^ This is because low medical density implies that each physician has less time to allocate to each patient and, therefore, he/she replaces time-consuming therapies with opioid analgesics. Important geographic variation in opioid prescribing is also found in the US, where the availability of physicians also represents an important predictor of opioid use [43]. Further discussion on the opioid epidemic in the US is carried out in “Opioid crisis”.

### Economic conditions, mortality, and health

A vast literature exists on the relationship between poverty, income, income inequality, and health outcomes. In this literature, there is a significant agreement on the existence of a positive relationship between poverty and poor health. Mackenbach et al. [41] perform a study comparing health inequalities across 22 European countries by considering data on mortality, self-reported health, age, sex, and socioeconomic status. They show that, in each selected country, mortality rates are more prominent among the least educated and those belonging to lower occupational classes. Worse self-reported health is also more prevalent in lower socioeconomic groups in all countries. In France, Heritage [35] studies the relationship between socioeconomic status and self-reported health. By controlling for age and gender, she shows that self-reported health is positively associated with income, education, and professional status. Benzeval and Judge [3] offer a brief review of 16 longitudinal studies on this topic. These works use various techniques to control for reverse causality, also called, in this specific context, health selection, referring to the idea that poor health limits the individual’s ability to work, thus exerting a negative impact on future earnings and income. Results suggest that the relationship running from poverty to health has to be considered causal. Finally, Pickett and Wilkinson [53] offer a review of the literature on income inequality and health. They find strong evidence that poor health outcomes are more prevalent in more unequal communities and argue that this evidence is convincing enough to conclude that the link between income inequality and health is causal.

While studies exploiting individual-level data typically find a positive relationship between low socioeconomic status and poor health outcomes, the literature analyzing linkages between business cycles, mortality, and health behavior is more controversial. Papers in this literature typically use aggregate measures, such as the unemployment rate, to proxy economic activity. Some authors uncover pro-cyclical effects, meaning that mortality decreases and physical health improves in periods of economic turndowns. Some others uncover counter-cyclical effects instead. Taken altogether, however, prior findings suggest that the relationship between macroeconomic fluctuations, mortality, and health largely depends on the country, the time period, and the cause of morbidity and mortality under analysis. Ogburn and Thomas [50] were the first to find simultaneous fluctuations of business cycles and overall mortality, infant mortality, and death rates from tuberculosis in the US. By using panel data covering a 20-year period, Buchmueller et al. [8] also find pro-cyclical effects in France, where the negative association with local unemployment is the strongest in case of deaths related to cardiovascular conditions and accidents. This study has been recently updated by Brüning and Thuilliez [7], who further test the robustness of their results using alternative econometric specifications. Their results differ from those in Buchmueller et al. [8], though, since they find no systematic relationship between the unemployment rate at the department level and mortality in France. Ruhm [56] offers a discussion of studies in this literature.

### Opioid crisis

By documenting an unexpected decline in life expectancy for middle-aged non-Hispanic white Americans during the last two decades, Case and Deaton [9, 10] first introduced the expression ‘deaths of despair’. They postulated that living in a disadvantaged socioeconomic environment induces individuals to consume more licit and illicit substances, including opioid painkillers, thus resulting in impoverished health outcomes and increased mortality. In the context of the US opioid crisis, a growing literature focuses on the relationship between the (over)use of prescription pills and labor-market outcomes. This line of research has found mixed results, especially concerning the direction of causality.

Some studies seem to corroborate the ‘deaths of despair’ hypothesis by suggesting that economic variables play an important role in fueling the epidemic. Hollingsworth et al. [37] show how macroeconomic fluctuations, as proxied by variations in the unemployment rate, are related to measures of opioid-related harm and how the latter increases in periods of economic distress. Similarly, Charles et al. [14] find that increasing unemployment in local manufacturing is positively associated with opioid use and deaths. Ghertner and Groves [27] claim that prescription opioid sales and opioid-related harm are more common in areas characterized by poor economic conditions, while Venkataramani et al. [65] find a significant positive association between automotive plant closures and opioid overdose mortality. O’Brien et al. [49] show that automation, the introduction of industrial robots in manufacturing, causes an increase in drug overdose deaths. In addition, this negative effect is stronger in areas with a larger supply of opioid pain relievers.

Other papers cast doubt on the assumption that economic impairment led to the opioid crisis and suggest that the causal link runs in the opposite direction. Krueger [38] shows how the decline in US labor force participation is positively associated with the increased use of opioid pain relievers. He estimates that, between 1999 and 2015, increased opioid prescriptions could be responsible for as much as 20% of the fall in labor force participation for males and 25% for females. Laird and Nielsen [39] also find prescription opioids responsible for declines in Denmark’s labor force participation and income. Based on the observation that shifts in the type of drugs (opioid analgesics versus illicit opioids) causing overdose deaths have been contextual to changes in the composition of deaths, Ruhm [56] concludes that the driving forces of the epidemic need not be found in worsening economic conditions, but should rather be linked to specific characteristics of the public health environment. As a consequence, he sustains that policy interventions aimed at improving economic prospects would have a limited impact, if any, and proposes instead to push more on remedies aimed at affecting the drug environment (such as prescription drug monitoring programs, development of abuse-deterrent drugs, and improved education for healthcare professionals). Currie et al. [19] focus on employment as a proxy for economic status and find ambiguous results. The authors conclude that the relationship between opioid use and instrumented employment is relatively weak and, hence, the roots of the crisis need to be found in reasons other than economic disruption. Finally, Currie and Schwandt [18] confirm that the relationship between labor-market opportunities and opioids is too weak to explain the magnitude of the opioid crisis in the US.

In addition to demand-side (economic) factors, the recent literature on the opioid crisis identifies a few additional elements related to the US epidemic, which are worth mentioning here.

First, the epidemic is partially due to the exponential increase in the number of prescriptions by physicians. Since 1986, the World Health Organization (WHO) has encouraged healthcare professionals to take pain treatment more seriously into account, and since 2001, the Joint Commission has invited physicians to consider pain as the ‘fifth vital sign’ for evaluating patients’ health. This, combined with the industry’s marketing effort, has eventually led to overprescribing opioids. Schnell [60] tries to rationalize this phenomenon and shows that physicians prescribe at least 20% more than what would be optimal.

Aggressive marketing by pharmaceutical companies is another primary ingredient of the epidemic. In the US, for example, Purdue Pharma (the manufacturer of OxyContin, a potent opioid analgesic) has been sued several times for distributing advertising material that overstated the benefits of opioids while understating their addiction risks (e.g., a promotional video distributed to general practitioners claimed that the risk of getting addicted to OxyContin was as low as 1%). Pharmaceutical companies’ marketing strategies have been shown to be effective in influencing physicians’ prescribing habits. For example, Hadland et al. [32, 33] show how direct-to-physician advertising of opioids is associated with increased prescribing and positively related to opioid overdoses. Fernandez and Zejcirovic [25] take a step further by uncovering the causal link between opioid product promotion to opioid overdose deaths.

Finally, over-consumption is exacerbated by the presence of a secondary black market and by patients’ specific behaviors (such as doctor shopping and pharmacy shopping).^8^ The National Survey on Drug Use and Health [5] reveals that, in 2016, 53% of individuals misusing opioid pain relievers obtained them from a friend or a relative (for free, by paying or stealing), 6% bought them from a drug dealer, and 1.5% prescriptions were given by more than one doctor. In this respect, Nordmann et al. [48] study the prevalence of doctor shopping in three French regions and observe that this practice is more prevalent in the region with the most unfavorable socioeconomic environment (in terms of poverty, unemployment, number of crimes), even though they do not offer an econometric analysis supporting this statement.

Compared to previous studies, the structure of the data provided by *OpenHealth* allows us to control for these (potential confounding) factors influencing prescription opioid use and better isolate the role of economic impairment.

## Institutional background

In France, the healthcare sector is highly regulated at the national level. In this section, we summarize the main features of the French health system. We discuss drug scheduling, pharmaceutical pricing, and reimbursement schemes, as well as health insurance coverage.

In France, drugs are classified into different lists according to the risks linked to their use. Scheduled medicines entail higher risks than non-scheduled ones. Medicines in ‘Liste 1’ are associated with higher risks than those in ‘Liste 2’. ‘Stupefiants’ stand for narcotics and are considered the most dangerous. Each list features different rules concerning the type and length of the prescription, the need to split the delivery, and the possibility of marketing the products through the Internet or media.^9^ Specifically, ‘Médicaments non listés’ (i.e., non-scheduled drugs) can be obtained at the pharmacy without a prescription and can be reimbursable or not. For both ‘Liste 1’ and ‘Liste 2’ drugs, a simple prescription form is required. However, while for the latter the prescription can be renewed, for the former, the prescription is usually non-renewable, and its duration cannot exceed 12 months. Finally, ‘Stupefiants’ need a special prescription form called ‘ordonnance sécurisée’. The prescription’s duration cannot exceed 28 days, but this term can vary between 7 and 28 days, depending on the active substance. All strong opioid products in our database are classified as ‘Stupefiants’. All mild opioid products belong to ‘Liste 1’, with the exception of 14 codeine products that remained available over the counter (OTC) until July 2017.

Pharmaceutical pricing and reimbursement schemes are also highly regulated in France. The procedure for a new drug to reach the market is articulated in several steps. First, the medicine must receive marketing authorization (Autorisation de Mise sur le Marché, AMM) by the European or national authorities.^10^ This marketing authorization is issued for a period of 5 years and is renewable.^11^ Conditional on receiving an AMM, the drug manufacturer may decide whether to apply for the medicine to be reimbursed by Social Security. If the pharmaceutical company does not apply for reimbursement, the product can be directly launched onto the market, and the patient has to pay its full price, which the company can freely set. If instead, the manufacturer requires reimbursement, the medicine is assessed by a Transparency Committee (Commission de la Transparence). Its task consists of evaluating the drug’s therapeutic benefit (Service Médical Rendu, SMR) and its added value relative to existing treatments (Amélioration du Service Medical Rendu, ASMR), and providing suggestions on the reimbursement rate.^12^ After this, negotiations are carried out between the drug manufacturer and an Economic Committee for Health Products (Comité Economique des Produits de Santé), composed of representatives of the Ministry of Health and the Ministry of Economy and Finance. The drug’s price is the result of these negotiations and depends mainly on the ASMR of the new drug.^13^ The National Healthcare Insurance Funds (Union Nationale des Caisses d’Assurance Maladie, UNCAM) eventually establishes the reimbursement rate, which varies according to the product’s medical benefit (SMR). The Health Ministry eventually decides whether to include the drug on the registry of reimbursable medicines. The duration of this inscription is 5 years,^14^ after which the Transparency Committee must reassess the drug. Once the described procedure is concluded, the new drug can be launched onto the market.

Finally, the National Health Insurance Scheme guarantees universal coverage: health insurance is compulsory for all those who reside in France for at least 3 months. This insurance covers around 70% of doctors’ fees (25 euros) and 80% of hospital costs. The reimbursement rate for most of the opioid pain relievers in our database is, instead, 65%. The remaining portion of these expenses represents out-of-pocket spending that can be either paid directly by the patient or through complementary insurance. However, around 90% of French residents own supplementary health insurance. In addition, all those whose income is below a certain threshold are entitled to receive the Universal Complementary Health Insurance (Couverture Maladie Universelle Complémentaire, CMU-C) or the State Medical Assistance (Aide au Paiement d’une Complémentaire Santé, ACS).^15^ The former provides free access to supplementary health insurance, while the latter covers 50% of the complementary insurance cost.

It is worth noticing that, while regulatory differences across regions may matter for narcotic consumption, our focus on France allows us to bypass this channel because regulations, policy interventions, and rules governing the healthcare system are centralized in France. There exist regional health agencies (‘Agences Régionales de Santé’) in charge of the management and efficient allocation of healthcare resources and services, as well as regional entities in charge of pharmacovigilance (‘Centres Régionaux de Pharmacovigilance’). However, these agencies do not have regulatory power and implement directives received by the central government.

## Data and summary statistics

Data for the set of variables used to describe the department’s socioeconomic status are downloaded from the INSEE (Institut National de la Statistique et des Études Économiques) and DREES (Direction de la Recherche, des Études, de l’Évaluation et des Statistiques) websites. Our variables are selected mainly based on the existing literature on the US opioid epidemic.

### Data sources

*Opioid retail sale data* The *OpenHealth* database contains information on opioid retail sales in France since 2008 in terms of consumer units sold, that is, the number of packs sold for each product. Sales data are provided at the national level and for each of the 94 French departments composing Metropolitan France.^16^ For our econometric analysis, we exploit firm-level annual data for each department from 2008 to 2017. For the descriptive analysis in “Nationwide consumption and substitution patterns”, we use aggregate annual data at the national level.

For each item, the database indicates the product’s denomination, the name of the pharmaceutical company marketing it, the number of packs sold, the number of pills in each pack, and the quantity of the active ingredient (in milligrams) contained in each pill. This allows us to compute the total quantity (in mg) sold of each product and convert this to the number of DDDs consumed, which is the metric recommended by the WHO for analyzing drug consumption. DDD means defined daily dose and is defined by the WHO as ‘the assumed average maintenance dose per day for a drug used for its main indication in adults’, that is, the amount (in mg) of an active ingredient that should be daily administered to an average weight adult (70 kg) for a drug’s main indication. Using this metric allows comparing consumption trends across different products as well as aggregating consumption data for different active ingredients. For the regression analysis of “Econometric analysis”, drug usage is measured in terms of number of DDDs per 1000 inhabitants by exploiting the following formula:

$$\begin{aligned} {\text {DU}} = \frac{n \times p \times {\text {mg/}}p \times 1000}{{\text {DDD}} \times h}, \end{aligned}$$

where DU denotes drug usage, *n* is the number of packs sold, *p* is the number of pills in a pack, mg/p is the number of milligrams per pill, and *h* is the number of inhabitants in the geographical area of interest. Finally, DDD refers to the official measure for each active substance as provided on the WHO website.^17^ When we discuss results at a more aggregate level for mild and strong opioids, DU is the sum of the DDDs consumed for each active ingredient in the set. In the descriptive analysis of “Nationwide consumption and substitution patterns”, we measure consumption in terms of number of DDDs consumed per 1000 inhabitants per day.

*Poverty and unemployment* The poverty rate is defined by the OECD (Organization for Economic Co-operation and Development) as the share of individuals living below the poverty line, usually set at 60% of the national median income.^18^ The poverty rate is analogously defined by the INSEE. The portion of individuals below the poverty line is computed based on the ‘disposable income per consumption unit (CU)’, that is, the disposable income per ‘adult equivalent’ in each household. This is calculated by dividing the household’s disposable income by the number of consumption units composing it. All persons attached to the same tax household have the same disposable income per CU. The number of consumption units in a household is, in turn, calculated as follows: one consumption unit for the first adult in the household, 0.5 CU for other people aged 14 or over, 0.3 CU for children younger than 14 years. For example, a couple without children counts for 1.5 CU, while a couple with two children under 14 years counts for 2.1 CU. This computation allows taking into account the economies of scale generated when living together.

Data for the unemployment rate are also provided by the INSEE. The unemployment rate is defined as the share of unemployed individuals divided by the active population.

*Population, age groups, and education* Population and age groups data are available for every year, while education data are only available for 1999, 2010, and 2015 (according to the censuses realized in these years).

The INSEE provides the number of individuals older than 16 years and no longer attending school (“population non-scolarisée”) in each education group, where each group refers to a different diploma level. The first group represents the portion of people with no diploma or with a DNB (“Diplôme National du Brevet”) awarded after completion of the first cycle of education. The second group represents the share of individuals holding a BEP (“Brevet d’Étude Professionnelle”) or CAP (“Certificat d’Aptitude Professionnelle”). These are obtained after completing 2 years of a professional high school (“Lycée”). The third group represents the share of people owning a “Baccalauréat” obtained after high school completion. The last group includes individuals with a “Diplôme d’Études Supérieures ”, that is, a university degree. Education data are interpolated as follows. First, we consider the difference in the number of individuals in each education group between two subsequent censuses, and we divide this number by the number of years between the two censuses to obtain an average annual variation. Next, the number of individuals in each education group is computed based on this annual variation for the years in which data are missing. For 2016 and 2017, we apply the same annual variation as for the years between 2010 and 2015. Finally, we divide the number of individuals in each education group by the department population each year to obtain the share of people in each group. For our analysis, we aggregate the first three education groups to obtain the share of individuals with, at most, a high school diploma. We also divide the number of individuals in each age group by the department’s population each year to obtain the share of people in a given age group.

*Population density as a proxy for rurality* To characterize departments as rural or urban, we use the population density, measured as the number of inhabitants (in thousands) per square kilometer, and with the understanding that a more densely populated department is ‘more urban’ than a less populated one. This choice is based on the observation that both the OECD and EUROSTAT classify geographical areas by employing a three-step approach, mainly based on population density. To construct our population density variable, we exploit population data and data on the area of each department (in square kilometers) provided by INSEE.

*Healthcare professionals* Data on general practitioners (GPs) are collected from DREES and expressed in terms of densities, that is, the number of GPs per 100,000 inhabitants.

### Summary statistics

Table 1 provides a list of variables used in “Econometric analysis”, together with summary statistics. The top two panels refer to opioid consumption, measured in DDDs per 1000 inhabitants, while the last panel reports economic and socio-demographic covariates. We provide summary statistics for the sets of mild and strong opioids as well as for each active ingredient separately. Since our dataset contains information at the firm level, these summary statistics represent means, standard deviations, minima and maxima across companies, (active substances), departments and time periods.^19^

**Table 1:** Summary statistics

Variables	No. of obs.	Mean	Std. dev.	Min	Max
*Mild opioids (DDDs per 1000 inh.)*					
Codeine	19,364	164.294	423.199	0	5095
Tramadol	25,662	168.646	415.07	0	7236.397
Total mild	45,026	166.774	418.586	0	7236.397
*Strong opioids (DDDs per 1000 inh.)*					
Oxycodone	1692	116.156	212.843	0	2982.157
Transdermal fentanyl	7708	39.199	89.177	0	1265.315
Transmucosal fentanyl	3384	14.236	13.756	.032	93.417
Oral morphine	3854	85.219	217.321	0	4752.454
Injectable morphine	3290	12.157	42.368	0	1470.359
Total strong	19,928	45.930	132.190	0	4752.454
*Socio-economic covariates*					
Poverty rate (%)	940	.143	.030	.073	.29
Unemployment rate (%)	940	.091	.019	.04	.155
Age 40–59 (%)	940	.273	.009	.243	.295
Age 60+ (%)	940	.260	.045	.147	.376
(Only) basic education (%)	940	.581	.062	.300	.687
Population density	940	0.571	2.469	0.015	21.347
GPs density	940	152.863	25.492	101.764	293.367

The average number of DDDs consumed per 1000 inhabitants is about 167 for mild opioids and 46 for strong opioids. However, the difference between the minimum and the maximum is staggering, ranging from 0 to 7236 for mild and from 0 to 4752 for strong opioids. This shows that there exists substantial variation in consumption across products, departments, and time periods, and this variation persists even when considering each active substance. A considerable part of this variation is undoubtedly due to nationwide increasing trends in opioid use (see “Nationwide consumption and substitution patterns”) as well as firm-level differences in sales. For example, in the Oxycodone market, the company Mundipharma^20^ has much larger market shares than its competitors. However, even neglecting this firm and time dimensions, variability in opioid use across departments persists. This may appear surprising, given the French ‘centralized culture’, according to which rules governing the healthcare system are invariant across geographical areas. This evidence suggests that national policies determining the availability of opioid medications are not the only ones responsible for opioid consumption and spark our interest in investigating the role played by the demand-side determinants of opioid use. This investigation is facilitated by the sizable variation in our economic and socio-demographic observables. For example, the average poverty rate in France is $14.3\%$ but ranges from a minimum of $7.3\%$ to a maximum of $29\%$. In the next section, we further discuss nationwide trends in prescription opioid use during the period 2008–2017. In “Econometric analysis”, we show that, even once nationwide trends and company-specific characteristics are taken into account, the relationship between economic status and opioid use remains significant.

## Nationwide consumption and substitution patterns

This section provides a descriptive analysis of sales trends and substitution patterns among different classes of analgesics in France from 2008 to 2017. Our description encompasses each active ingredient individually and, at a more aggregate level, the sets of mild and strong opioids. The distinction between mild and strong opioids is made according to WHO’s three-step ladder for treatment of chronic pain, which classifies analgesics as (i) non-opioids, such as non-steroidal anti-inflammatory drugs (NSAIDs), paracetamol and ibuprofen; (ii) mild opioids, such as codeine combinations and tramadol (alone or in combination);^21^ (iii) strong opioids, such as oxycodone, fentanyl and morphine.^22^

**Fig. 1:**
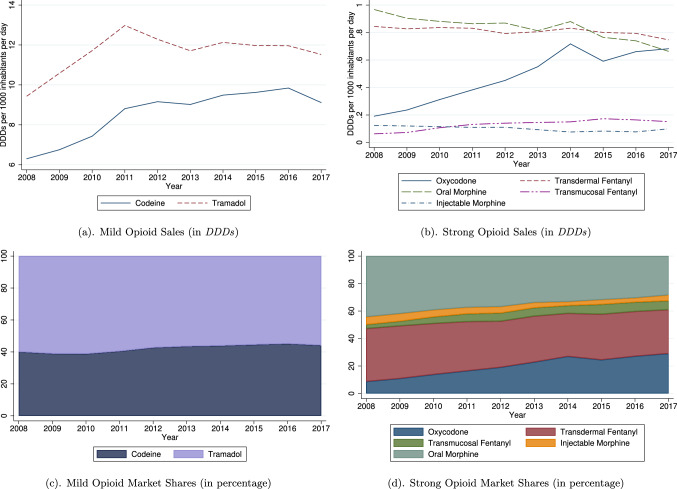
Opioid analgesic sales in France: trends and market shares Sources: OpenHealth, Authors’ calculation

**Fig. 2:**
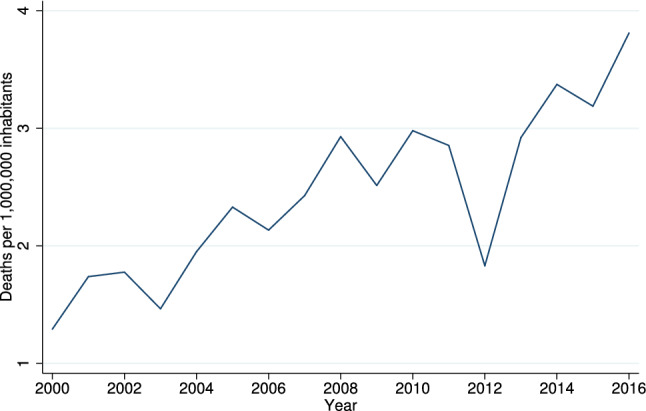
Opioid-related deaths in France between 2000 and 2016 Sources: CépiDc-Inserim database; authors’ calculations

**Fig. 4:**
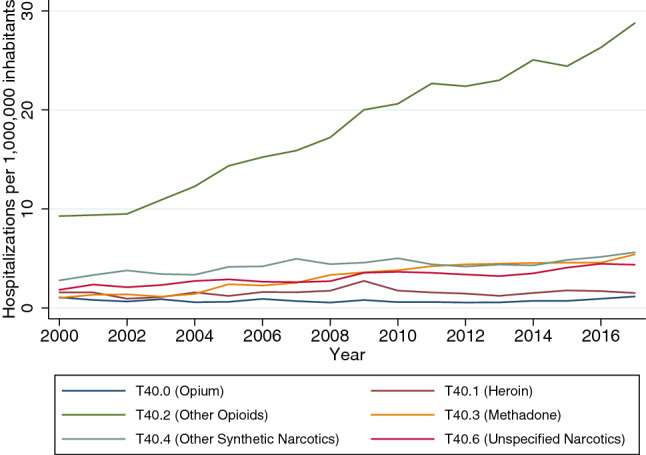
Opioid-related hospitalizations in France between 2000 and 2017 Sources: ScanSanté database; authors’ calculations

The most commonly used analgesics are mild opioids. Panel (a) in Fig. 1 shows that tramadol, alone or in combination, is more frequently administered than codeine, which is only available in combination. Consumption of both these active ingredients increases during the 10 years. Codeine consumption rises by 45%, from 6.3 DDDs per 1000 inhabitants per day in 2008 to 9.1 in 2017. Tramadol retail sales also increase, even though at a slower pace, from 9.4 to 11.5 DDDs (a 22% increase). Tramadol peaks in 2011 and, then, slightly declines between 2011 and 2013. By contrast, codeine consumption keeps rising during the whole period under examination. Overall, mild opioid sales rise by 31%. Panel (c) in Fig. 1 shows that the shares of codeine and tramadol remained stable over time.

In the group of strong opioids (panels (b) and (d)), oral morphine is the most widely used analgesic, even though its sales fall from 0.97 to 0.66 DDDs. Injectable morphine slightly decreases from 0.12 to 0.10. Transdermal fentanyl is the second most commonly used strong opioid. Its sales remain approximately constant over time, even though they slightly decline: consumption for this substance drops by 11%. Oxycodone consumption, instead, exhibits a spectacular increase, reaching and even overcoming Morphine in 2017. Its sales rise from 0.19 to 0.68 DDDs, a 257% variation. Trends also show that oxycodone retail sales slowed down in 2014 and 2015, a period during which generics started to enter the oxycodone market. Before this date, the market was a monopoly, where the only manufacturer was Mundipharma. Panel (d) in Fig. 1 reveals that the fall in oxycodone sales between 2014 and 2015 has been mainly absorbed by fentanyl (in part by transdermal, in part by transmucosal).^23^ Finally, transmucosal fentanyl registers the second highest variation rate, from 0.06 to 0.15 DDDs: a 142% increase. Overall, strong opioid consumption increases by approximately 7%, from 2.19 DDDs in 2008 to 2.34 in 2017. Panel (d) further suggests that Morphine is increasingly being replaced by Oxycodone and, to a lesser extent, by fentanyl. This pattern may raise some concerns since fentanyl and oxycodone are claimed to be stronger than morphine.

In summary, this descriptive analysis highlights the following stylized facts: mild opioids are the most consumed in France; tramadol is the most widely used; morphine is the most commonly administered among strong opioids; oxycodone experiences the largest expansion in sales. These findings are particularly meaningful if we consider that, according to the Agence Nationale de Sécurité du Médicament et des Produits de Santé [2], tramadol, morphine, and oxycodone were the substances most frequently involved in intoxications in 2016. The high consumption of fentanyl may also raise concerns due to its strength (10 times stronger than morphine).

## Econometric analysis

In this section, we assess the responsiveness of local per capita opioid analgesic sales to changes in economic and socio-demographic indicators. To do this, we run a series of panel regressions of per capita opioid use on socioeconomic determinants in 94 French departments (Metropolitan France) for the years 2008–2017. We mainly focus on the relationship between opioid consumption and economic conditions as proxied by the poverty rate.

### Econometric specification

Our unit of observation is a company–substance–department–year combination. In Tables 2, 3, 4, 5 that follow, we progressively add controls to isolate the effect of poverty on opioid consumption. Our first specification in column (1) of each table is:

1 $$\begin{aligned} {\text {log}} (Y_{csdt}+1) = \beta _{0}+\beta _{1} X_{{{d}}t}+\eta _{{c}}+\gamma _{{r}}+\delta _{t}+\lambda _{rt}+u_{csdt}, \end{aligned}$$

where $Y_{csdt}$ is consumption for product(s) by company *c* containing the active ingredient *s* in department *d* and year *t*. We use a log-level specification, in which consumption is logged, while the right-hand side variables are kept in levels since they are already expressed in percentage terms.^24^ In the above equation, $X_{dt}$ exclusively contains our main variable of interest, the poverty rate. We further include company ($\eta _{c}$), region ($\gamma _{r}$), year ($\delta _{t}$) and region-by-year ($\lambda _{rt}$) fixed effects. This allows us to exploit variation across departments while controlling for time-invariant differences across products/companies, for national-level shocks or policies affecting opioid use and for time-varying regional characteristics. Indeed, in France, there exist regional health agencies (‘Agences Régionales de Santé’) in charge of the management and efficient allocation of healthcare resources and services as well as regional entities in charge of pharmacovigilance (‘Centres Régionaux de Pharmacovigilance’). Hence, one concern may be that the availability of healthcare resources is correlated with the prevalence of poverty in the department and influences opioid use at the same time.

In columns (2)–(3)–(4) of each table, we substitute the regional fixed effects by department ($\alpha _{d}$) fixed effects to control for department-specific time-invariant characteristics:

2 $$\begin{aligned} {\text {log}} (Y_{csdt}+1)= \beta _{0}+\beta _{1} X_{{dt}}+\eta _{c}+\alpha _{d}+\delta _{t}+u_{csdt}, \end{aligned}$$

where $X_{dt}$ exclusively contains the poverty rate in column (2). We add the unemployment rate in column (3) and the remaining controls in column (4). These include other department socio-demographic characteristics (age groups, GPs density, population density, and share of individuals with high school diploma) that have been shown in the previous literature to correlate with opioid consumption. Indeed, prior studies on the opioid crisis in the USA have shown that opioid use is more prevalent among middle-aged individuals and people with less than college education. We further control for the population density as a proxy for the ‘rurality’ of the department since previous research has shown that the opioid epidemic has strongly hit rural communities. Finally, we include the density of doctors in the department to control for the prevalence of doctor-shopping and for the presence of frequent prescribers, two additional factors indicated by the literature as partially responsible for the opioid epidemic in the US. We focus on general practitioners (GPs) since, in France, slightly less than 90% of opioid prescribers are GPs [2]. Silhol et al. [62] also show that, in France, GPs practicing in the so-called ‘medical deserts’ (geographical areas characterized by low doctors’ density) tend to prescribe more opioid analgesics. Finally, medical density can also be viewed as a proxy for accessibility to healthcare services.

In columns (5) and (6), we include company-by-year ($\phi _{ct}$) and company-by-department ($\theta _{cd}$) fixed effects, which allow us to control for company-specific factors that vary over time and across departments, respectively:

3 $$\begin{aligned} {\text {log}} (Y_{csdt}+1)= \beta _{0}+\beta _{1} X_{dt}+\eta _{c} +\alpha _{d}+\delta _{t}+\phi _{ct}+\theta _{cd}+u_{csdt}. \end{aligned}$$

This is important since the aggressive marketing strategies implemented by some pharmaceutical companies have been identified as one of the main determinants of the US opioid epidemic, while Goupil et al. [30] provide evidence that French general practitioners are also sensitive to pharmaceutical promotion. These fixed effects allow us to control for differences in pharmaceutical spending across companies as well as for companies’ marketing strategies targeting specific departments.^25^ Besides promotional activity, this allows us to control for some important observed and unobserved determinants of demand, including brand equity and potential discrepancies in prices of products by different companies.^26^ Finally, column (6) also introduces the education variable.

In all regressions, $\beta _{1}$ is the vector of parameters to be estimated and $u_{csdt}$ is an idiosyncratic error term, which is clustered at the department level to adjust for potential serial correlation. Moreover, we weight observations in regressions by the department population to correct for heteroskedasticity due to different population sizes [63]. For $\beta _{1}$ to be an unbiased estimator, our covariates, including the fixed effects, should control for all potential confounding factors. We claim that our data and econometric setup allow us to account for a vast pool of factors highlighted by the literature as being related to opioid use. Finally, each regression is run for the sets of mild and strong opioids as well as for each active ingredient separately. This allows us to further control for differences in advertising strategies across products within the same company, and for differences in prices across active ingredients and companies. We can additionally uncover different patterns in the way each substance relates to socioeconomic indicators.

### Baseline results

Detailed regression results for mild and strong opioids are reported in Tables 2 and 3.

In Table 2 (mild opioids), the poverty rate coefficient is always positive and significant at the 1% level, except in column (6), where it is significant at 5%. Its magnitude goes from 1.9 in column (1), with regional fixed effects, to 5.3 in column (2), with department fixed effects. This shows that neglecting department-specific factors introduces significant downward bias. In columns (2) to (6), the magnitude of the estimated coefficients ranges between 5.2 and 6, meaning that when the poverty rate increases by one percentage point, mild opioid use rises by 5–6%. Unemployment is never significant, instead. One concern may be that this is due to some degree of collinearity with the poverty variable. However, running the same regressions as in Table 2 by excluding the poverty rate yields similar results as well as regressions that exclude the unemployment rate. In addition, considering the log unemployment rate produces substantively identical results.^27^ The coefficient for the middle-age group is also positive and significant at the 10% confidence level. A one percentage point increase in the share of individuals in this age group is associated with a 14% increase in the use of mild opioid analgesics. Mild opioid use also seems more common in rural areas, since the coefficient associated with the population density is negative and significant at the 1% confidence level. A 100 additional inhabitants per square kilometer would yield approximately a 4% increase in mild opioid consumption. The coefficient associated to education is positive and significant at the 10% level, whereas the availability of general practitioners does not seem to play a role.^28^ Finally, the adjusted $R^2$ suggests that our covariates explain an important share of the variation in opioid analgesic use. Its magnitude increases the most going from column (4) to column (5), that is, when we add the company–department and company–period fixed effects. This suggests that pharmaceutical promotion, as well as other determinants of the demand previously discussed, also play a relevant role in explaining opioid analgesic use.

**Table 2:** Regression results—aggregate mild opioid consumption

Dependent variable: (log) mild opioids	(1)	(2)	(3)	(4)	(5)	(6)
Poverty	1.914***	5.256***	5.666***	6.027***	5.975***	5.362**
rate	(0.372)	(1.850)	(1.900)	(2.243)	(2.236)	(2.285)
Unemployment			− 3.340	− 0.214	− 0.176	− 1.319
rate			(5.103)	(3.944)	(3.940)	(3.780)
Age (40–59)				14.258*	14.622*	14.426*
				(7.976)	(8.066)	(7.619)
Age (60+)				3.569	3.558	− 3.338
				(4.248)	(4.300)	(5.204)
GPs density				− 0.001	− 0.002	− 0.002
				(0.002)	(0.002)	(0.002)
Population				− 0.404***	− 0.400***	− 0.378***
density				(0.100)	(0.100)	(0.086)
(Only) basic						8.342*
education						(4.664)
Company FE	$\checkmark$	$\checkmark$	$\checkmark$	$\checkmark$	$\checkmark$	$\checkmark$
Region FE	$\checkmark$					
Year FE	$\checkmark$	$\checkmark$	$\checkmark$	$\checkmark$	$\checkmark$	$\checkmark$
Region-by-year FE	$\checkmark$					
Department FE		$\checkmark$	$\checkmark$	$\checkmark$	$\checkmark$	$\checkmark$
Company-by-year FE					$\checkmark$	$\checkmark$
Company-by-department FE					$\checkmark$	$\checkmark$
Adjusted $R^2$	0.757	0.761	0.761	0.761	0.833	0.834
*N*	45,026	45,026	45,026	45,026	44,932	44,932

Robust standard errors clustered at the department level in parentheses

Regressions are weighted by the local population size

$$^{*}p<0.1,\, ^{**}p< 0.05,\, ^{***}p < 0.01$$

Table 3 shows that the relationship between strong opioids and poverty is much weaker than for mild opioids. The estimated coefficient is smaller in magnitude compared to the one in Table 2, and significant in the specifications of columns (1) and (4) only. Individuals in the middle age do not seem to significantly consume more strong analgesics than individuals in other age groups. The rurality of the department matters, instead. The coefficient associated with the population density is significant and similar in magnitude to the one obtained for mild opioids. This result is mostly driven by transdermal fentanyl, transmucosal fentanyl, and oral morphine (see Appendix 3). The coefficient associated to education is again positive and significant. Finally, the same considerations made above about the unemployment rate continue to hold for strong opioids.

**Table 3:** Regression results—aggregate strong opioid consumption

Dependent variable: (log) strong opioids	(1)	(2)	(3)	(4)	(5)	(6)
Poverty	0.936**	2.188	1.923	4.243*	3.906	3.217
rate	(0.427)	(1.694)	(1.756)	(2.419)	(2.390)	(2.413)
Unemployment			2.215	2.644	1.468	0.016
rate			(4.615)	(4.682)	(4.981)	(4.863)
Age (40–59)				6.822	5.770	5.685
				(8.722)	(9.092)	(8.508)
Age (60+)				3.392	1.971	− 7.469
				(5.103)	(4.825)	(5.194)
GPs density				0.002	0.002	0.002
				(0.002)	(0.002)	(0.002)
Population				− 0.372***	− 0.424***	− 0.397***
Density				(0.138)	(0.134)	(0.113)
(Only) basic						11.552***
education						(4.246)
Company FE	$\checkmark$	$\checkmark$	$\checkmark$	$\checkmark$	$\checkmark$	$\checkmark$
Region FE	$\checkmark$					
Year FE	$\checkmark$	$\checkmark$	$\checkmark$	$\checkmark$	$\checkmark$	$\checkmark$
Region-by-year FE	$\checkmark$					
Department FE		$\checkmark$	$\checkmark$	$\checkmark$	$\checkmark$	$\checkmark$
Company-by-year FE					$\checkmark$	$\checkmark$
Company-by-department FE					$\checkmark$	$\checkmark$
Adjusted $R^2$	0.755	0.759	0.759	0.759	0.822	0.822
*N*	19,923	19,928	19,928	19,928	19,928	19,928

Robust standard errors clustered at the department level in parentheses

Regressions are weighted by the local population size

$$^{*}p<0.1,\, ^{**}p< 0.05,\, ^{***}p < 0.01$$

Table 4 reports the estimated poverty rate coefficients *only* for each active substance separately. Each row refers to an active ingredient, whereas each column contains the same set of covariates as in Tables 2 and 3. Appendix 3 further provides the full results for each active ingredient. Table 4, as well as Tables 11 and 12 in Appendix 3, show that results closely mirror those in Table 2 for mild opioids. Codeine and tramadol behave similarly in the way they relate to socioeconomic factors.

**Table 4:** Regression results—poverty rate estimates by active substance

Dependent variable: (in logs)	(1)	(2)	(3)	(4)	(5)	(6)
*Mild opioids*						
Codeine	2.003***	5.850***	6.313***	6.669***	6.606***	5.865**
	(0.412)	(2.034)	(2.064)	(2.420)	(2.409)	(2.458)
Tramadol	1.848***	4.639***	4.991***	5.335**	5.604**	5.094**
	(0.346)	(1.665)	(1.732)	(2.071)	(2.137)	(2.188)
*Strong opioids*						
Oxycodone	1.056	− 0.675	− 1.391	− 2.738	1.229	0.716
	(0.765)	(3.423)	(3.724)	(4.682)	(4.971)	(5.042)
Transdermal	1.692***	4.236***	4.541***	4.825***	3.885**	3.687**
fentanyl	(0.320)	(1.275)	(1.337)	(1.655)	(1.571)	(1.574)
Transmucosal	1.687***	5.509***	5.900***	5.874***	4.418**	4.160**
fentanyl	(0.320)	(1.607)	(1.631)	(1.960)	(1.746)	(1.759)
Oral	0.079	0.873	0.916	1.257	1.159	− 0.351
morphine	(0.632)	(3.395)	(3.590)	(4.195)	(4.689)	(4.876)
Injectable	− 0.773	5.339	4.426	13.493***	11.889**	9.431*
morphine	(1.205)	(3.487)	(3.551)	(4.759)	(4.895)	(4.971)

Robust standard errors clustered at the department level in parentheses

Regressions are weighted by the local population size

$$^{*}p<0.1,\, ^{**}p< 0.05,\, ^{***}p < 0.01$$

For strong opioids instead, results in Table 4 show that transdermal and transmucosal fentanyl, as well as injectable morphine, exhibit a positive and significant association with the poverty rate, while oxycodone and oral morphine do not. Hence, the presence of oxycodone and oral morphine attenuates the estimated coefficient for poverty in the regressions for all strong opioids in Table 3. Among the strong opioids, oxycodone and oral morphine behave differently, compared to the other substances, in the way they relate to economic hardship. This suggests that other factors, such as company-specific and department-specific characteristics, play a major role in determining the consumption of these active ingredients.

### Alternative measures of poverty

We perform here the same analysis as in the previous subsection by employing alternative measures of local poverty. We consider the poverty rate, defined as the share of individuals living with less than 50% of the national median income, as well as the Gini coefficient, measuring income inequality. The Gini coefficient takes values between 0 and 1, with higher values corresponding to higher income inequality. We additionally consider regressions where the poverty rate is lagged to, at least partially, control for potential endogeneity issues related to reverse causality.

**Table 5:** Regression results—alternative measures of poverty

	(1)	(2)	(3)	(4)	(5)	(6)
*(Log) mild opioids*						
Poverty 60%	1.914***	5.256***	5.666***	6.027***	5.975***	5.362**
	(0.372)	(1.850)	(1.900)	(2.243)	(2.236)	(2.285)
Poverty 50%	2.615***	6.482**	6.997**	6.709**	6.658**	5.853*
	(0.586 )	(2.646)	(2.784)	(3.014)	(3.017)	(3.166)
Gini	− 1.076**	8.804***	8.808***	7.766***	7.731***	7.644***
coefficient	(0.513)	(2.431)	(2.410)	(2.490)	(2.490)	(2.571)
Lagged poverty	1.915***	5.018***	5.316***	5.155***	5.266***	5.065***
	(0.353)	(1.498)	(1.559)	(1.698)	(1.711)	(1.723)
*(Log) strong opioids*						
Poverty 60%	0.936**	2.188	1.923	4.243*	3.906	3.217
	(0.427)	(1.694)	(1.756)	(2.419)	(2.390)	(2.413)
Poverty 50%	1.362**	2.464	2.053	4.662	3.324	2.457
	(0.645)	(2.200)	(2.319)	(3.137)	(3.152)	(3.276)
Gini	− 0.099	3.263	3.354	5.393*	6.457**	6.526**
coefficient	(0.575)	(2.326)	(2.369)	(2.917)	(2.876)	(2.831)
Lagged poverty	0.991**	1.997	2.015	3.323*	2.943	2.678
	(0.431)	(1.405)	(1.481)	(1.879)	(1.883)	(1.885)

Robust standard errors clustered at the department level in parentheses

Regressions are weighted by the local population size

$$^{*}p<0.1,\, ^{**}p< 0.05,\, ^{***}p < 0.01$$

Table 5 contains the results. The upper panel refers to mild opioids and the lower panel to strong opioids. To facilitate comparison, we report results from the previous subsection as well as the estimated coefficients for our alternative measures of poverty. Each row refers to a different poverty measure, whereas each column contains the same set of covariates as in Tables 2, 3, 4.

Table 5 largely confirms the results from the previous subsection. For the set of mild opioids, the estimated coefficients for poverty (at 50%), Gini, and lagged poverty rate are always positive and significant. The only exception is for the Gini coefficient estimate in the specification of column (1). This confirms that failing to take into account department-specific factors introduces significant downward bias. Interestingly, the estimates for poverty 50% are always larger in magnitude than those for poverty 60%. For instance, if we consider the specification of column (3), this means that a one percentage point increase in the share of people living with less than 50% of the national median income is associated with a 7% increase in mild opioid consumption, while the same variation in the share of individuals living with less than 60% of the national median income yields a 5.7% increase in mild opioids. This suggests a role for the intensity of poverty since an increase in the share of ‘very’ poor individuals leads to a larger increase in opioid analgesic use. Coefficient estimates for the Gini coefficient show that income inequality also represents an important predictor of opioid use.

For the set of strong opioids, the estimated coefficient for poverty 50% is positive but significant only in the specification of column (1), while the lagged poverty rate is significant in columns (1) and (4). Gini coefficient estimates, instead, are positive and significant in columns (4) to (6), but remain smaller compared to the estimates for mild opioids.

Finally, the estimated coefficients for the remaining controls, which we do not show here, are similar to those of “Baseline results”.^29^

Results for the Gini coefficient require further discussion. As mentioned in “Economic conditions, mortality, and health”, the existence of an association between income inequality and health is not new to the literature. Some authors have explained this association in terms of the so-called ‘absolute income’ or ‘poverty’ hypothesis, according to which income inequality and health are not directly related, and the observed correlation is the result of income inequality actually being a proxy for poverty [22]. If we believe in this hypothesis, the observed correlation between income inequality and opioid use in our data confirms our findings regarding the relationship between poverty and opioid analgesic consumption, especially for mild opioids. Notice, however, that even though the Gini coefficient and the poverty rate are positively correlated in our data, they do not perfectly coincide, and, indeed, income inequality seems a better predictor for strong opioid use compared to the poverty rate.

### Alternative specifications

We now discuss the robustness of our results to alternative econometric specifications. We consider regressions omitting the population weighting, adding a department-specific time trend, and combining the full set of FEs with region–year fixed effects. We also provide results for regressions using the number of DDDs consumed in the population in levels (rather than in logs) and estimating the model via Poisson pseudo-maximum likelihood (PPML).^30^ For this analysis, we focus on the most complete specifications of equation (3), corresponding to columns (5) and (6) in Tables 2, 3, 4, 5.

Columns (1) and (2) of Table 6 show that, once the population weighting is removed, the magnitude of the coefficients associated with the poverty rate decreases for both mild and strong opioids, and estimates are no longer statistically significant for mild opioids. At the same time, standard errors are 19–24% larger than before. This suggests that using population weights in this context actually helps correct for heteroskedasticity and obtain more precise estimates [63].

The remaining specifications produce much larger and more significant estimates for strong opioids compared to Table 3. This suggests that taking into account trending factors and regional characteristics may be important when analyzing strong opioid use.

**Table 6:** Regression results—alternative specifications

	No weighting		Department-specific trend		Region-year FEs		Poisson (PPML)	
	(1)	(2)	(3)	(4)	(5)	(6)	(7)	(8)
*Mild opioids*								
Poverty	3.773	3.448	3.845**	3.730**	5.519*	5.102	4.673	4.288
rate	(2.735)	(2.725)	(1.685)	(1.735)	(3.196)	(3.288)	(3.331)	(3.290)
*Strong opioids*								
Poverty	1.448	1.262	6.291***	6.290***	9.313***	8.916**	12.348**	11.747**
rate	(2.953)	(2.950)	(1.928)	(1.935)	(3.522)	(3.616)	(5.246)	(5.243)

Robust standard errors clustered at the department level in parentheses

$$^{*}p<0.1,\, ^{**}p< 0.05,\, ^{***}p < 0.01$$

### Discussion

The results presented in “Baseline results” and “Alternative measures of poverty” are consistent with previous findings in the US and France described in “Literature review”. The prevalence of poverty at the local level is significantly related to the use of opioid analgesics in France, especially for mild opioids. In addition, this finding is consistent across alternative definitions of poverty. In contrast to Ruhm [56], the effect of our economic proxy becomes sometimes larger and more significant when controlling for possible fixed and time-varying confounding factors. However, in line with Ruhm [56], we find that the relationship between poverty and opioids is less relevant for strong opioids.

The unemployment rate, instead, does not seem to play a role in fueling opioid consumption in France: the coefficient associated with unemployment is not statistically different from zero. This contrasts with previous studies in the US, showing that unemployment is positively related to opioid use and abuse (e.g., [37]). However, our study confirms prior findings by Ruhm [55] and Brüning and Thuilliez [7], showing that, especially during the last decades, mortality is weakly related or unrelated to macroeconomic conditions in the US and France, respectively.

Poverty is not the same as unemployment, a variable used in other studies to proxy economic status. Even though unemployment and poverty are often positively correlated, being unemployed does not necessarily mean being poor (and reciprocally), and the French system provides considerable monetary support to unemployed individuals who can avoid falling into a poverty trap. Indeed, we find (consistent with Currie et al. [19]) that unemployment is not—but that poverty is—significantly related to opioid sales. Therefore, unemployment does not necessarily represent a good proxy for economic disruption in France, and the type of despair mentioned by Case and Deaton [9, 10] is more likely to be a poor individual than a (temporary) unemployed one.

While regulatory differences across regions may matter for narcotic consumption, our focus on France allows us to bypass this channel because regulations, policy interventions, and rules governing the healthcare system are centralized in France. We additionally control for potential differences across regions in the management of healthcare resources as well as important supply-side factors, such as pharmaceutical advertising. Yet, our analysis shows that poverty plays a significant role in driving opioid analgesic sales.

By including socio-demographic indicators as controls, we also complement previous research. The incidence of pain across age groups in France, for instance, is studied by Hadjiat et al. [31]. They find that chronic pain is more prevalent among adults aged between 45 and 64. In the US, the major part of opioid-related overdose deaths in 2015 occurred among individuals aged between 25 and 55 [12], while Case and Deaton [9, 10] suggest that the opioid epidemic is an important contributor to the increase in mortality among middle-aged non-Hispanic whites.^31^

Furthermore, the US opioid epidemic seems to have affected more heavily rural communities [29]. For example, the Government Accountability Office [26] explains that the first reports of widespread abuse and diversion of OxyContin appeared in rural areas. Cicero et al. [17] show that prescription opioid misusers are most commonly white, reside in suburban or rural areas and have less than a college education.

We improve on the existing literature in at least three respects. First, compared to studies using measures of opioid-related harm as outcome variables (e.g., opioid-related deaths), we observe sales for each opioid product on the market. This allows us to exclusively focus on opioids meant for medical use, thus avoiding contamination from illicit opioid use in our dependent variable. Moreover, we can perform an analysis substance by substance, thus observing which specific substances drive our results. Second, we exploit firm-level data, which allows us to control for important supply-side factors, such as pharmaceutical companies’ marketing, and better isolate the impact of economic hardship. Finally, we are the first to perform this type of studies in Europe.

## Conclusions and policy implications

Economic hardship, and specifically the prevalence of poverty at the local level, may positively affect opioid analgesic use through a few potential channels. First, according to the so-called ‘deaths of despair’ hypothesis [9, 10], individuals living in disadvantaged conditions are prone to consume more licit and illicit substances, including prescription opioids, in search of emotional relief and to evade a reality made of social stigma and exclusion. Indeed, there exists evidence showing that individuals living in poverty are more likely to abuse drugs and develop a dependence [1]. Second, it is known that poverty represents a risk factor for mental illnesses [58] such as depression and anxiety, and that individuals with these conditions tend to experience pain more frequently and intensively compared with the general population [20]. This is because the regions of the brain that modulates responses to pain are also those that generate feelings of anxiety and depression. Third, it is possible that physicians in contact with individuals in poverty are less careful about pure medical indications and prescribe opioids more frequently. As mentioned in “Geographical variation in healthcare utilization”, a few studies find that there exists significant variation in practice style not only across but also within physicians [45]. By exploiting prescription data, Chen et al. [15] show that physicians practicing in the most deprived areas in the UK prescribe significantly more opioid analgesics. A thorough investigation of this channel requires individual-level prescription data that were not available for the current study and should be the focus of future research.

The natural implication of this is that intensified demand-side interventions, such as state safety net programs, social inclusion measures and, more in general, investment in social infrastructure, would help a great deal to reduce the incidence of opioid abuse and opioid-related harm. As shown in O’Brien et al. [49], increased safety net program generosity has the potential to reduce drug overdose deaths. As of 1st June 2009, France approved a reform of the social system aimed at introducing a new instrument, called ‘Revenu de Solidarité Active (RSA)’, to provide stronger return-to-employment incentives while increasing the long-term disposable income of low-income households. Analyzing the impact of this reform on opioid analgesic consumption and opioid-related harm represents an interesting topic for future research.

At the same time, supply-side policy interventions also undoubtedly play a crucial role in addressing opioid abuse. Indeed, the significant discrepancies in mortality rates between France and the US^32^ may largely be attributed to profoundly different regulatory systems and medical cultures. For example, in France, it is strictly forbidden to advertise prescription-only medicines publicly, pharmaceutical companies are not allowed to provide free samples for narcotic medications, and the bureaucratic burden associated with opioid prescription is much heavier. A less liberal medical culture may also make the difference: European doctors are more conservative and more reluctant to use opioids than their US counterparts. According to Nguemeni Tiako et al. [47], the centralized nature of the French healthcare system has also facilitated the collaboration between addiction facilities, NGOs, regional health agencies, and the Ministry of Health that permitted an efficient response to the needs of individuals with opioid use disorders (OUD). Their joint goal is, among others, to facilitate (free) access to addiction treatments and harm-reduction services, such as syringe exchange and supervised drug consumption sites. This national network played a crucial role, especially amid the COVID-19 pandemic, by pushing to increase access to naloxone, buprenorphine, and methadone, facilitate access to medications for opioid use disorders and provide social support and housing for people who use drugs. Improving physicians’ education and providing incentives for them to substitute pharmacological treatments with available alternatives (e.g., acupuncture) is also important, given their gatekeeper role, since opioid analgesics can only be obtained through a prescription. GPs should be aware that patients living in disadvantaged areas are most at risk. Prior research in the US shows that when a physician discovers that one of his/her patients died from an opioid overdose, he/she subsequently prescribes fewer opioid analgesics [23]. Moreover, physicians’ who received their degrees from the best medical schools also prescribe significantly fewer opioid painkillers [61]. This shows that doctors are sensitive to the type of information and training they receive.

We conclude that both socioeconomic aspects and regulatory frameworks are crucial for reducing opioid-related harm. Policies aimed at fighting the epidemic should not translate in an out-out between improving socioeconomic status or enhancing the regulatory environment, but should rather view these as complementary for addressing the same crisis.

Finally, our analysis of the French context highlights the interplay between national policies and local economic prospects: any new regulation imposed at the national level is likely to trigger heterogeneous responses across geographical regions and pharmaceutical companies because the final consumers (the patients) will react differently depending on their economic status and the producers (the firms) may, then, have an incentive to revise their marketing strategies. These topics represent the focus of future research.

## Figures and Tables

**Fig. 3:**
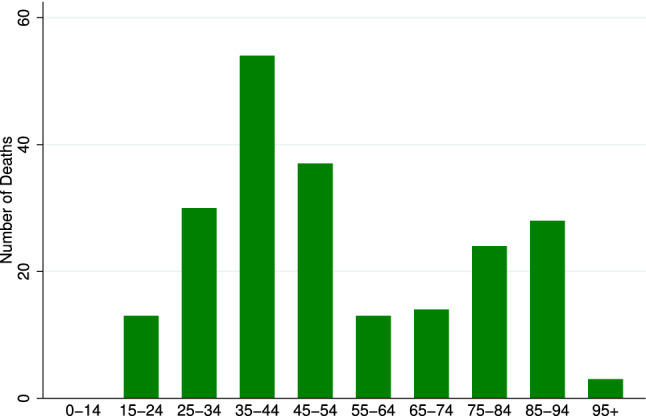
Opioid-related deaths by age group in 2014 Sources: CépiDc-Inserim database; authors’ calculations

## Appendix 1: Opioid analgesic sales data

### The OpenHealth database

The *OpenHealth* data are obtained via extrapolation from a panel of pharmacies in Metropolitan France, excluding Corsica. This panel includes 12,562 pharmacies in total in 2022, yielding a coverage rate of 62% (the coverage rate was 46% in 2017). Data from these pharmacies are collected on a daily basis. In addition, their extrapolation model is adapted to the number of pharmacies transmitting the data. This means that comparability of results is guaranteed, even though the number of transmitters evolves over time.

The extrapolation method is based on a multi-stratified model, where pharmacies are first stratified according to several criteria, among which their overall turnover, their geographical location and the characteristics of their location. A ‘representativeness’ weight is then applied to each stratum. These weights are calculated in real time by considering the ratio between the number of pharmacies transmitting the data and the actual number of pharmacies respecting the stratum’s criteria. These extrapolation coefficients are eventually applied to sales ticket data to obtain the total sales for the geographical area of interest. The OpenHealth pharmacy reference database includes all pharmacies as obtained from the source FINESS (Fichier National des Etablissements Sanitaires et Sociaux). The reference database is updated daily and in real time. This grants a good quality of information for the stratification criteria.^33^

### Opioid products and companies

This appendix contains information about the opioid analgesics used for the analysis in this paper and the pharmaceutical companies marketing them. Table 7 lists the active substances, the name of each product, the route of administration and the available dosages.^34^ Table 8 provides the name of the companies selling mild opioids and specifies whether sales data for each company are available for the whole period between 2008 and 2017. Table 9 contains the same information for strong opioids.

**Table 7:** Opioid analgesics

Active ingredient	Product denomination	Administration route	Dosages
Codeine and paracetamol	Algicalm, algisedal, codoliprane, compralgyl, dafalgan codeine, gaosedal, klipal codeine, lindilane, claradol codeine, doliprane codeine, and generics	Oral	20, 25, 30, 50 mg
Codeine and ibuprofen	Antarene codeine	Oral	30, 60 mg
Codeine, paracetamol, and cafeine	Migralgine, prontalgine	Oral	20 mg
Codeine, paracetamol, and aspirine	Novacetol	Oral	10 mg
Codeine, aspirine, and cafeine	Sedaspir	Oral	20 mg
Tramadol	Biodalgic, topalgic, contramal, monoalgic, monocrixo, monotramal, orozamudol, predalgic, takadol, zamudol, zumalgic, trasedal, and other generics	Oral	50–100–150–200–300 mg and 100 mg/ml
Tramadol and paracetamol	Ixprim, zaldiar and generics	Oral	37.5 mg
Tramadol and dexketoprofene	Skudexum	Oral	75 mg
Oxycodone	Oxycontin, oxynorm, oxynormoro and generics	Oral	5–10–15–20–30–40–60–80–120 mg
Morphine	Actiskenan, aguettant, sevredol, skenan, oramorph, moscontin, kapanol	Oral	5 mg/ml, 10 mg/5 ml, 20 mg/1 ml, 30 mg/5 ml, 100 mg/5 ml and 5–10–20–30–50–60–100–200 mg
Morphine	Lavoisier, renaudin, aguettant, cooper, meram	Injectable	0.1–1–10–20–40–50 mg/ml, 50 mg/5 ml, 100 mg/5 ml
Fentanyl	Durogesic, matrifen and generics	Transdermal	12–25–50–75–100 $\upmu$g/h
Fentanyl	Abstral, actiq, breakyl, effentora, instanyl, pecfent, recivit	Transmucosal	67–100–133–200–267–300–400–533—600-800–1200–1600 $\upmu$g

**Table 8:** Pharmaceutical companies (mild opioids)

Active ingredient	Company	Entry	Exit
Codeine + paracetamol	Cooperation Pharmaceutique Francaise	< 2008	On the market
	Arrow Generiques	< 2008	On the market
	Bayer Sante Familiale	< 2008	On the market
	Biogaran	< 2008	On the market
	Bristol Mayers	< 2008	On the market
	Cristers	2009	On the market
	EG Laboratoire	< 2008	On the market
	Gifrer Barbezat	< 2008	On the market
	Grunenthal	< 2008	On the market
	Merck Medication Familial sas	< 2008	On the market
	Mylan	< 2008	On the market
	Mylan Medical sas	< 2008	On the market
	Pierre Fabre Medicaments	< 2008	On the market
	Sandoz sas	< 2008	On the market
	Sanofi Aventis France	< 2008	On the market
	Teva Sante	< 2008	On the market
Codeine + ibuprofen	Elerte	2011	On the market
Codeine (combinations)	Pharmastra	< 2008	On the market
	Bride	< 2008	On the market
	Johnson Johnson	< 2008	On the market
	Boehringer-Ingelheim France	< 2008	On the market
Tramadol	Arrow Generiques	< 2008	On the market
	Actavis	2011	On the market
	Biocodex	< 2008	On the market
	Biogaran	< 2008	On the market
	Cristers	2014	On the market
	EG Laboratoire	< 2008	On the market
	Elerte	< 2008	2015
	Evolupharm	2013	On the market
	Expanscience	< 2008	On the market
	GNR Pharma	2008	2012
	Grunenthal	< 2008	On the market
	Laboratoire X.O	< 2008	On the market
	Mylan	< 2008	On the market
	Mylan Medical sas	< 2008	On the market
	Qualimed	< 2008	On the market
	RPG Ranbaxy Pharm. Generiq.	2013	On the market
	Sandoz sas	< 2008	On the market
	Sanofi Aventis France	2008	On the market
	Sanofi Zentiva	< 2008	On the market
	Teva Sante	< 2008	On the market
	Therabel Lucien Pharma	< 2008	On the market
	Zydus France sas	< 2008	On the market
Tramadol + paracetamol	Arrow Generiques	2013	On the market
	Biogaran	2013	On the market
	Cristers	2013	On the market
	EG Laboratoire	2014	On the market
	Evolupharm	2013	On the market
	Grunenthal	< 2008	On the market
	KRKA Pharma	2013	On the market
	Mylan	2013	On the market
	Mylan Medical sas	2014	On the market
	Pharma Reference PHR Lab	2013	2017
	RPG Ranbaxy Pharm. Gen.	2013	On the market
	Sandoz sas	2013	On the market
	Sanofi Zentiva	2013	On the market
	Teva Sante	2013	On the market
	Zydus France sas	2013	On the market
Tramadol + dexketoprofene	Menarini France	2017	On the market

**Table 9:** Pharmaceutical companies (strong opioids)

Active ingredient	Company	Entry	Exit
Oxicodone	Mundipharma	< 2008	On the market
	Arrow Generiques	2017	On the market
	Mylan	2014	On the market
	EG Laboratoire	2015	On the market
Transdermal fentanyl	Janssen Cilag sa	< 2008	On the market
	Takeda	2009	On the market
	Arrow Generiques	2010	On the market
	Biogaran	2009	On the market
	EG Laboratoire	2010	On the market
	Mylan	2013	On the market
	RPG Ranbaxy Pharm. Generiq.	2013	On the market
	Sandoz sas	2009	On the market
	Teva Sante	< 2008	On the market
	Zentiva	2009	On the market
Transmucosal fentanyl	Kyowa Kirin Pharma	2009	On the market
	Teva Sante	< 2008	On the market
	Mylan Medical sas	2013	On the market
	Takeda	2010	On the market
	Grunenthal	2014	On the market
Oral morphine	Ethypharm	< 2008	On the market
	Aguettant	< 2008	2012
	GSK	< 2008	2013
	Mundipharma	< 2008	On the market
	Kyowa Kirin Pharma	< 2008	On the market
Injectable morphine	Aguettant	< 2008	On the market
	Cooperation Pharmaceutique Francaise	< 2008	On the market
	Chaix et du Marais	< 2008	On the market
	Renaudin	2013	On the market

## Appendix 2: Opioid-related harm

This section provides a descriptive analysis of opioid-related hospitalizations and deaths in France. “The International Classification of Disease (ICD-10) system” describes the International Classification of Disease System, “Opioid-related death” analyses mortality data, and “Opioid-related hospitalizations” deals with hospitalization data.

### The International Classification of Disease (ICD-10) system

In the International Classification of Disease System, causes of death are coded based on the Underlying Cause of Death (UCD).^35^ The WHO defines the Underlying Cause of Death as ‘the disease or injury which initiated the train of morbid events leading directly to death, or the circumstances of the accident or violence which produced the fatal injury’.

Drug poisonings are coded as ‘external causes of morbidity and mortality’ in Chapter XX and identified by ICD-10 codes X40–X44, X60–X64, X85, and Y10–Y14. Moreover, Chapter V identifies mental and behavioral disorders due to psychoactive substance use in codes F01–F99. For opioid-related deaths, we use codes F11, identifying deaths due to mental and behavioral disorders related to opioid use, X42, identifying deaths due to accidental poisoning by narcotics and psychodysleptics [hallucinogens] and exposure to these products, X62, identifying deaths due to auto-intoxication through narcotics and psychodysleptics [hallucinogens] and exposure to these products, Y12, identifying deaths due to poisoning by narcotics and psychodysleptics [hallucinogens] and exposure to these products, when the intention is not known.

When the underlying cause of death is poisoning (Chapter XX), the death certificate should also report the substance(s) involved using codes T36 to T50 in Chapter XIX.^36^ For opioid-related hospitalizations, we use the code T40 concerning poisonings by narcotics and psychodysleptics [hallucinogens], where the fourth digit indicates the specific substance causing the poisoning: T40.0 for opium; T40.1 for heroin; T40.2 for other opioids (codeine, morphine, natural and semisynthetic opioids); T40.3 for methadone; T40.4 for other synthetic narcotics (pethidine, other synthetic opioids) and T40.6 for other and unspecified narcotics.

### Opioid-related death

Mortality data related to opioid consumption are extracted from the *Centre d’Épidémiologie sur les Causes Médicales de Décès* [13] database on all causes of death in France from 1979 to 2016. In this database, the causes of death are coded and classified according to the International Classification of Diseases (ICD-10). In order to identify deaths related to problematic use of opioids, we selected the codes F11, X42, X62 and Y12.^37^

Figure 2 depicts the trend in opioid-related deaths between 2000 and 2016, where the *y*-axis reports the number of deaths per 1,000,000 inhabitants. For this graph, we restrict attention to data concerning accidental deaths only (codes F11 and X42). In France, opioid-related deaths rose from 1.3 to 3.8 per one million inhabitants, which is a 192% increase over the 2000–2016 period and almost 5 deaths per week in 2016.^38^

Figure 3 describes the partition of deaths across age groups in 2014. The majority of opioid-related deaths concerns individuals between 35 and 44 years old. In particular, 25% of deaths involve individuals in this age group, while 17.13% pertain to those in the 45–54 group so that these two groups alone account for 42.13% of all opioid-related deaths. If we further include the 25–34 age group, which accounts for 13.89% of deaths, we conclude that about 56% of opioid deaths are related to individuals between 25 and 54 years old. This is consistent with findings in the US and with our regression results, according to which individuals between 40 and 59 years old tend to consume more prescription opioids than the rest of the population.

Despite this consistency, these results need to be interpreted with caution because, from these mortality data, we cannot determine to which specific substance the death is related. This means that we cannot infer how many deaths are related to prescription opioids. Hence, we tried a set of regressions of opioid-related deaths on prescription opioid sales, by using the following specification:

$$\begin{aligned} {\text {Deaths}}_{dt} = \beta _{0}+\beta _{1} ({\text {log}}) {\text {PrescriptionOpioids}}_{dt}+\alpha _{d}+\delta _{t}+u_{dt}. \end{aligned}$$

Results are reported in Table 10. We use mortality data for the years 2008–2016 and the 94 departments composing Metropolitan France. The independent variable is the natural logarithm of opioid analgesic sales while, for the dependent variable, we use levels, rather than logs, since this variable takes on zero value for some departments in some years. In addition, the dependent variable changes across columns of Table 10, depending on which codes are considered. Specifically, the first column considers deaths due to accidental poisoning only (codes F11 and X42), the second adds deaths due to auto-intoxication (codes F11, X42, and X62), the third column includes accidental deaths plus deaths for which the intention is not known (codes F11, X42, and Y12) and the last column considers all deaths. This dependent variable measures the number of deaths per 100,000 inhabitants. Finally, each regression includes department and year fixed effects while the error term is clustered at the department level to correct for potential serial correlation. The estimated coefficients are always positive and significant, indicating that increases in sales of opioid pain relievers are associated with increases in opioid-related deaths. Moreover, the estimated coefficients are larger in magnitude and more significant, when we include auto-intoxication cases. This result may be an indicator that prescription opioids are employed by individuals with suicidal intentions. This issue has been raised by a few authors in the USA [51], pointing to its relevance for policy purposes.

**Table 10:** Regression results—opioid-related deaths

	(1)	(2)	(3)	(4)
	Deaths	Deaths	Deaths	Deaths
	F11-X42	F11-X42-X62	F11-X42-Y12	F11-X42-X62-Y12
Log (all opioids)	0.107**	0.131***	0.104**	0.129***
	(0.042)	(0.043)	(0.042)	(0.043)
Adjusted $R^2$	0.192	0.206	0.195	0.207
*N*	846	846	846	846

Robust standard errors clustered at the department level in parentheses

Each regression includes department and year fixed effects and is weighted by the department population $$^{*} p<0.1$$, $$^{**} p < 0.05$$, $$^{***} p < 0.01$$

### Opioid-related hospitalizations

Data for opioid-related hospitalizations are extracted from the *ScanSanté* database, made available by the ‘Agence Technique de l’Information sur l’Hospitalisation’ (ATIH) (ScanSanté, Agence Technique de l’Information sur l’Hospitalisation (ATIH), (2019)). We select data for which the main diagnosis is intoxication related to opioid use, identified by the International ICD-10 system with the codes T40.0 (opium), T40.1 (heroin), T40.2 (other opioids), T40.3 (methadone), T40.4 (other synthetic opioids), T40.6 (unspecified narcotics).

Figure 4 below depicts the trend in opioid-related hospitalizations between 2000 and 2017, where the *y*-axis reports the number of hospitalizations per 1,000,000 inhabitants. In France, opioid-related hospitalizations potentially due to prescription pills (codes T40.0, T40.2, T40.4, and T40.6) rose from about 15 to 39.91 per one million inhabitants, which is a 166.6% increase over the 2000–2017 period and approximately seven hospitalizations per day in 2017.^39^

## Appendix 3: Regression results by active ingredient

See Tables 11, 12, 13, 14, 15, 16, 17.

**Table 11:** Regression results—codeine combinations

Dependent variable: (log) codeine	(1)	(2)	(3)	(4)	(5)	(6)
Poverty	2.003***	5.850***	6.313***	6.669***	6.606***	5.865**
rate	(0.412)	(2.034)	(2.064)	(2.420)	(2.409)	(2.458)
Unemployment			− 3.834	− 0.481	− 0.448	− 1.782
rate			(5.431)	(4.249)	(4.240)	(4.034)
Age (40–59)				15.871*	15.703*	15.407*
				(8.735)	(8.712)	(8.168)
Age (60+)				3.796	3.765	− 4.060
				(4.715)	(4.699)	(5.766)
GPs density				− 0.001	− 0.001	− 0.002
				(0.003)	(0.003)	(0.003)
Population				− 0.435***	− 0.433***	− 0.406***
density				(0.120)	(0.119)	(0.103)
(Only) basic						9.458*
education						(5.029)
Company FE	$\checkmark$	$\checkmark$	$\checkmark$	$\checkmark$	$\checkmark$	$\checkmark$
Region FE	$\checkmark$					
Year FE	$\checkmark$	$\checkmark$	$\checkmark$	$\checkmark$	$\checkmark$	$\checkmark$
Region-by-year FE	$\checkmark$					
Department FE		$\checkmark$	$\checkmark$	$\checkmark$	$\checkmark$	$\checkmark$
Company-by-year FE					$\checkmark$	$\checkmark$
Company-by-department FE					$\checkmark$	$\checkmark$
Adjusted $R^2$	0.942	0.946	0.946	0.947	0.993	0.993
*N*	19,364	19,364	19,364	19,364	19,364	19,364

Robust standard errors clustered at the department level in parentheses

Regressions are weighted by the local population size

$$^{*} p<0.1,\, ^{**} p< 0.05,\, ^{***} p < 0.01$$

**Table 12:** Regression results—tramadol (alone or in combinations)

Dependent variable: (log) tramadol	(1)	(2)	(3)	(4)	(5)	(6)
Poverty	1.848***	4.639***	4.991***	5.335**	5.604**	5.094**
rate	(0.346)	(1.665)	(1.732)	(2.071)	(2.137)	(2.188)
Unemployment			− 2.844	0.017	0.065	− 0.889
rate			(4.756)	(3.647)	(3.731)	(3.604)
Age (40–59)				12.906*	14.595*	14.461*
				(7.320)	(7.717)	(7.357)
Age (60+)				3.363	3.403	− 2.582
				(3.835)	(4.096)	(4.905)
GPs density				− 0.002	− 0.002	− 0.002
				(0.002)	(0.002)	(0.002)
Population				− 0.369***	− 0.373***	− 0.354***
density				(0.095)	(0.095)	(0.085)
(Only) basic						7.246
education						(4.471)
Company FE	$\checkmark$	$\checkmark$	$\checkmark$	$\checkmark$	$\checkmark$	$\checkmark$
Region FE	$\checkmark$					
Year FE	$\checkmark$	$\checkmark$	$\checkmark$	$\checkmark$	$\checkmark$	$\checkmark$
Region-by-year FE	$\checkmark$					
Department FE		$\checkmark$	$\checkmark$	$\checkmark$	$\checkmark$	$\checkmark$
Company-by-year FE					$\checkmark$	$\checkmark$
Company-by-department FE					$\checkmark$	$\checkmark$
Adjusted $R^2$	0.793	0.796	0.796	0.796	0.937	0.937
*N*	25,662	25,662	25,662	25,662	25,568	25,568

Robust standard errors clustered at the department level in parentheses

Regressions are weighted by the local population size

$$^{*} p<0.1,\, ^{**} p< 0.05,\, ^{***} p < 0.01$$

**Table 13:** Regression results—oxycodone

Dependent variable: (log) oxycodone	(1)	(2)	(3)	(4)	(5)	(6)
Poverty	1.056	− 0.675	− 1.391	− 2.738	1.229	0.716
rate	(0.765)	(3.423)	(3.724)	(4.682)	(4.971)	(5.042)
Unemployment			5.388	9.484	2.629	1.724
rate			(12.083)	(11.832)	(11.000)	(10.911)
Age (40–59)				− 3.470	0.679	0.483
				(14.828)	(15.097)	(14.611)
Age (60+)				-16.248*	5.857	− 0.104
				(9.016)	(7.986)	(10.546)
GPs density				0.001	0.005	0.005
				(0.005)	(0.004)	(0.004)
Population				− 0.950***	− 0.646	− 0.628
density				(0.242)	(0.409)	(0.399)
(Only) basic						7.223
education						(9.513)
Company FE	$\checkmark$	$\checkmark$	$\checkmark$	$\checkmark$	$\checkmark$	$\checkmark$
Region FE	$\checkmark$					
Year FE	$\checkmark$	$\checkmark$	$\checkmark$	$\checkmark$	$\checkmark$	$\checkmark$
Region-by-year FE	$\checkmark$					
Department FE		$\checkmark$	$\checkmark$	$\checkmark$	$\checkmark$	$\checkmark$
Company-by-year FE					$\checkmark$	$\checkmark$
Company-by-department FE					$\checkmark$	$\checkmark$
Adjusted $R^2$	0.860	0.871	0.871	0.872	0.947	0.947
*N*	1692	1692	1692	1692	1598	1598

Robust standard errors clustered at the department level in parentheses

Regressions are weighted by the local population size

$$^{*}p<0.1,\, ^{**}p< 0.05,\, ^{***}p < 0.01$$

**Table 14:** Regression results—transdermal fentanyl

Dependent variable: (log) transdermal fentanyl	(1)	(2)	(3)	(4)	(5)	(6)
Poverty	1.692***	4.236***	4.541***	4.825***	3.885**	3.687**
rate	(0.320)	(1.275)	(1.337)	(1.655)	(1.571)	(1.574)
Unemployment			− 2.627	− 0.191	− 0.136	− 0.747
rate			(3.921)	(3.194)	(3.079)	(3.055)
Age (40–59)				8.137	6.053	6.175
				(6.076)	(5.748)	(5.631)
Age (60+)				1.742	1.616	− 2.809
				(3.201)	(2.997)	(3.506)
GPs density				− 0.001	− 0.001	− 0.001
				(0.002)	(0.002)	(0.002)
Population				− 0.338***	− 0.321***	− 0.315***
density				(0.099)	(0.122)	(0.119)
(Only) basic						5.485*
education						(3.221)
Company FE	$\checkmark$	$\checkmark$	$\checkmark$	$\checkmark$	$\checkmark$	$\checkmark$
Region FE	$\checkmark$					
Year FE	$\checkmark$	$\checkmark$	$\checkmark$	$\checkmark$	$\checkmark$	$\checkmark$
Region-by-year FE	$\checkmark$					
Department FE		$\checkmark$	$\checkmark$	$\checkmark$	$\checkmark$	$\checkmark$
Company-by-year FE					$\checkmark$	$\checkmark$
Company-by-department FE					$\checkmark$	$\checkmark$
Adjusted $R^2$	0.877	0.882	0.882	0.882	0.993	0.993
*N*	7708	7708	7708	7708	7708	7708

Robust standard errors clustered at the department level in parentheses

Regressions are weighted by the local population size

$$^{*}p<0.1,\, ^{**}p< 0.05,\, ^{***}p < 0.01$$

**Table 15:** Regression results—transmucosal fentanyl

Dependent variable: (log) transmucosal fentanyl	(1)	(2)	(3)	(4)	(5)	(6)
Poverty	1.687***	5.509***	5.900***	5.874***	4.418**	4.160**
rate	(0.320)	(1.607)	(1.631)	(1.960)	(1.746)	(1.759)
Unemployment			− 3.326	− 0.283	− 0.302	− 1.065
rate			(4.499)	(3.474)	(3.195)	(3.139)
Age (40–59)				12.126*	9.156	9.282
				(6.988)	(6.395)	(6.169)
Age (60+)				2.589	2.574	− 3.052
				(3.718)	(3.271)	(3.761)
GPs density				− 0.001	− 0.001	− 0.001
				(0.002)	(0.002)	(0.002)
Population				− 0.335***	− 0.299***	− 0.289***
density				(0.079)	(0.108)	(0.102)
(Only) basic						6.966*
education						(3.725)
Company FE	$\checkmark$	$\checkmark$	$\checkmark$	$\checkmark$	$\checkmark$	$\checkmark$
Region FE	$\checkmark$					
Year FE	$\checkmark$	$\checkmark$	$\checkmark$	$\checkmark$	$\checkmark$	$\checkmark$
Region-by-year FE	$\checkmark$					
Department FE		$\checkmark$	$\checkmark$	$\checkmark$	$\checkmark$	$\checkmark$
Company-by-year FE					$\checkmark$	$\checkmark$
Company-by-department FE					$\checkmark$	$\checkmark$
Adjusted $R^2$	0.881	0.889	0.889	0.889	0.990	0.990
*N*	3384	3384	3384	3384	3384	3384

Robust standard errors clustered at the department level in parentheses

Regressions are weighted by the local population size

$$^{*} p<0.1,\, ^{**} p< 0.05,\, ^{***} p < 0.01$$

**Table 16:** Regression results—oral morphine

Dependent variable: (log) oral morphine	(1)	(2)	(3)	(4)	(5)	(6)
Poverty	0.079	0.873	0.916	1.257	1.159	− 0.351
rate	(0.632)	(3.395)	(3.590)	(4.195)	(4.689)	(4.876)
Unemployment			− 0.364	3.806	7.156	5.088
rate			(7.269)	(6.114)	(6.930)	(6.794)
Age (40–59)				12.999	18.897	17.879
				(15.665)	(17.946)	(17.161)
Age (60+)				− 0.484	0.707	− 11.549
				(9.530)	(12.010)	(14.186)
GPs Density				− 0.002	− 0.002	− 0.003
				(0.004)	(0.004)	(0.004)
Population				− 0.860**	− 0.709*	− 0.636*
density				(0.352)	(0.365)	(0.331)
(Only) basic						14.810
education						(10.222)
Company FE	$\checkmark$	$\checkmark$	$\checkmark$	$\checkmark$	$\checkmark$	$\checkmark$
Region FE	$\checkmark$					
Year FE	$\checkmark$	$\checkmark$	$\checkmark$	$\checkmark$	$\checkmark$	$\checkmark$
Region-by-year FE	$\checkmark$					
Department FE		$\checkmark$	$\checkmark$	$\checkmark$	$\checkmark$	$\checkmark$
Company-by-year FE					$\checkmark$	$\checkmark$
Company-by-department FE					$\checkmark$	$\checkmark$
Adjusted $R^2$	0.917	0.924	0.924	0.924	0.957	0.957
*N*	3849	3854	3854	3854	3854	3854

Robust standard errors clustered at the department level in parentheses

Regressions are weighted by the local population size

$$^{*} p<0.1,\, ^{**} p< 0.05,\, ^{***} p < 0.01$$

**Table 17:** Regression results—injectable morphine

Dependent variable: (log) injectable morphine	(1)	(2)	(3)	(4)	(5)	(6)
Poverty	− 0.773	5.339	4.426	13.493***	11.889**	9.431*
rate	(1.205)	(3.487)	(3.551)	(4.759)	(4.895)	(4.971)
Unemployment			7.438	− 1.042	− 1.147	− 5.506
rate			(8.730)	(8.361)	(9.254)	(8.890)
Age (40–59)				12.211	3.913	2.980
				(16.558)	(17.093)	(15.501)
Age (60+)				12.900*	8.292	− 17.862*
				(7.506)	(7.609)	(9.607)
GPs density				0.014***	0.014***	0.013***
				(0.004)	(0.004)	(0.004)
Population				0.088	0.005	0.097
density				(0.323)	(0.301)	(0.268)
(Only) basic						31.598***
education						(8.287)
Company FE	$\checkmark$	$\checkmark$	$\checkmark$	$\checkmark$	$\checkmark$	$\checkmark$
Region FE	$\checkmark$					
Year FE	$\checkmark$	$\checkmark$	$\checkmark$	$\checkmark$	$\checkmark$	$\checkmark$
Region-by-year FE	$\checkmark$					
Department FE		$\checkmark$	$\checkmark$	$\checkmark$	$\checkmark$	$\checkmark$
Company-by-year FE					$\checkmark$	$\checkmark$
Company-by-department FE					$\checkmark$	$\checkmark$
Adjusted $R^2$	0.594	0.633	0.633	0.635	0.759	0.761
*N*	3290	3290	3290	3290	3290	3290

Robust standard errors clustered at the department level in parentheses

Regressions are weighted by the local population size

$$^{*} p<0.1,\, ^{**} p< 0.05,\, ^{***} p < 0.01$$

## Appendix 4: Additional regression results

See Tables 18, 19, 20, 21.

**Table 18:** Regression results—log unemployment rate (mild opioids)

Dependent variable: (log) mild opioids	(1)	(2)	(3)	(4)	(5)	(6)
Poverty	1.914***	5.256***	5.559***	6.180***	6.119***	5.315**
rate	(0.372)	(1.850)	(1.889)	(2.250)	(2.245)	(2.292)
(Log) Unemployment			− 0.409	− 0.160	− 0.150	− 0.162
rate			(0.458)	(0.346)	(0.344)	(0.347)
Age (40–59)				14.208*	14.566*	14.571*
				(7.903)	(8.001)	(7.588)
Age (60+)				3.548	3.535	− 3.188
				(4.260)	(4.313)	(5.152)
GPs density				− 0.001	− 0.001	− 0.002
				(0.002)	(0.002)	(0.002)
Population				− 0.394***	− 0.390***	− 0.371***
density				(0.103)	(0.103)	(0.089)
(Only) basic						8.131*
education						(4.721)
Company FE	$\checkmark$	$\checkmark$	$\checkmark$	$\checkmark$	$\checkmark$	$\checkmark$
Region FE	$\checkmark$					
Year FE	$\checkmark$	$\checkmark$	$\checkmark$	$\checkmark$	$\checkmark$	$\checkmark$
Region-by-year FE	$\checkmark$					
Department FE		$\checkmark$	$\checkmark$	$\checkmark$	$\checkmark$	$\checkmark$
Company-by-year FE					$\checkmark$	$\checkmark$
Company-by-department FE					$\checkmark$	$\checkmark$
Adjusted $R^2$	0.757	0.761	0.761	0.761	0.833	0.834
*N*	45,026	45,026	45,026	45,026	44,932	44,932

Robust standard errors clustered at the department level in parentheses

Regressions are weighted by the local population size

$$^{*} p<0.1,\, ^{**} p< 0.05,\, ^{***} p < 0.01$$

**Table 19:** Regression results—log unemployment rate (strong opioids)

Dependent variable: (log) strong opioids	(1)	(2)	(3)	(4)	(5)	(6)
Poverty	0.936**	2.188	2.150	4.618*	4.214*	3.283
rate	(0.427)	(1.694)	(1.704)	(2.380)	(2.353)	(2.374)
(Log) Unemployment			0.048	0.107	− 0.026	− 0.054
rate			(0.404)	(0.413)	(0.412)	(0.413)
Age (40–59)				6.420	5.520	5.653
				(8.697)	(9.116)	(8.560)
Age (60+)				3.406	1.968	− 7.494
				(5.156)	(4.855)	(5.194)
GPs density				0.002	0.002	0.002
				(0.002)	(0.002)	(0.002)
Population				− 0.369**	− 0.417***	− 0.393***
density				(0.142)	(0.136)	(0.115)
(Only) basic						11.575***
education						(4.312)
Company FE	$\checkmark$	$\checkmark$	$\checkmark$	$\checkmark$	$\checkmark$	$\checkmark$
Region FE	$\checkmark$					
Year FE	$\checkmark$	$\checkmark$	$\checkmark$	$\checkmark$	$\checkmark$	$\checkmark$
Region-by-year FE	$\checkmark$					
Department FE		$\checkmark$	$\checkmark$	$\checkmark$	$\checkmark$	$\checkmark$
Company-by-year FE					$\checkmark$	$\checkmark$
Company-by-department FE					$\checkmark$	$\checkmark$
Adjusted $R^2$	0.755	0.759	0.759	0.759	0.821	0.822
*N*	19,923	19,928	19,928	19,928	19,928	19,928

Robust standard errors clustered at the department level in parentheses

Regressions are weighted by the local population size

$$^{*} p<0.1,\, ^{**} p< 0.05,\, ^{***} p < 0.01$$

**Table 20:** Regression results—squared GPs density (mild opioids)

Dependent variable: (log) mild opioids	(1)	(2)	(3)	(4)	(5)	(6)
Poverty	1.914***	5.256***	5.666***	6.366***	6.311***	5.659**
rate	(0.372)	(1.850)	(1.900)	(2.209)	(2.200)	(2.276)
Unemployment			− 3.340	1.357	1.383	0.391
rate			(5.103)	(4.094)	(4.092)	(3.874)
Age (40–59)				14.107*	14.534*	14.278*
				(7.947)	(8.045)	(7.550)
Age (60+)				4.182	4.162	− 3.957
				(4.323)	(4.377)	(5.062)
GPs density				0.009	0.009	0.012*
				(0.007)	(0.007)	(0.007)
GPs density (squared)				− 2.78 $\times$ $10^{-5}$**	− 2.74 $\times$ $10^{-5}$**	− 3.41 $\times$ $10^{-5}$**
				(1.4 $\times$ $10^{-5}$)	(1.38 $\times$ $10^{-5}$)	(1.51 $\times$ $10^{-5}$)
Population				− 0.250**	− 0.253**	− 0.192
density				(0.125)	(0.124)	(0.122)
(Only) basic						9.997**
education						(4.590)
Company FE	$\checkmark$	$\checkmark$	$\checkmark$	$\checkmark$	$\checkmark$	$\checkmark$
Region FE	$\checkmark$					
Year FE	$\checkmark$	$\checkmark$	$\checkmark$	$\checkmark$	$\checkmark$	$\checkmark$
Region-by-year FE	$\checkmark$					
Department FE		$\checkmark$	$\checkmark$	$\checkmark$	$\checkmark$	$\checkmark$
Company-by-year FE					$\checkmark$	$\checkmark$
Company-by-department FE					$\checkmark$	$\checkmark$
Adjusted $R^2$	0.757	0.761	0.761	0.761	0.834	0.834
*N*	45,026	45,026	45,026	45,026	44,932	44,932

Robust standard errors clustered at the department level in parentheses

Regressions are weighted by the local population size

$$^{*} p<0.1,\, ^{**} p< 0.05,\, ^{***} p < 0.01$$

**Table 21:** Regression results—squared GPs density (strong opioids)

Dependent variable: (log) strong opioids	(1)	(2)	(3)	(4)	(5)	(6)
Poverty	0.936**	2.188	1.923	4.380*	4.015*	3.344
rate	(0.427)	(1.694)	(1.756)	(2.430)	(2.383)	(2.412)
Unemployment			2.215	3.425	2.106	0.951
rate			(4.615)	(4.977)	(5.326)	(5.151)
Age (40–59)				6.683	5.711	5.583
				(8.735)	(9.092)	(8.484)
Age (60+)				3.682	2.197	− 7.800
				(5.121)	(4.831)	(5.112)
GPs density				0.007	0.006	0.009
				(0.007)	(0.007)	(0.007)
GPs density (squared)				− 1.46 $\times$ $10^{-5}$	− 1.17 $\times$ $10^{-5}$	− 1.92 $\times$ $10^{-5}$
				(1.58 $\times$ $10^{-5}$)	(1.45 $\times$ $10^{-5}$)	(1.52 $\times$ $10^{-5}$)
Population				− 0.287*	− 0.361**	− 0.291**
Density				(0.165)	(0.147)	(0.136)
(Only) basic						12.411***
education						(4.403)
Company FE	$\checkmark$	$\checkmark$	$\checkmark$	$\checkmark$	$\checkmark$	$\checkmark$
Region FE	$\checkmark$					
Year FE	$\checkmark$	$\checkmark$	$\checkmark$	$\checkmark$	$\checkmark$	$\checkmark$
Region-by-year FE	$\checkmark$					
Department FE		$\checkmark$	$\checkmark$	$\checkmark$	$\checkmark$	$\checkmark$
Company-by-year FE					$\checkmark$	$\checkmark$
Company-by-department FE					$\checkmark$	$\checkmark$
Adjusted $R^2$	0.755	0.759	0.759	0.759	0.822	0.822
*N*	19,923	19,928	19,928	19,928	19,928	19,928

Robust standard errors clustered at the department level in parentheses

Regressions are weighted by the local population size

$$^{*} p<0.1,\, ^{**} p< 0.05,\, ^{***} p < 0.01$$

## Footnotes

1 ‘L’addiction aux opiacés, première cause de mort par overdose en France’, Le Monde (15th October, 2018).

2 Ginsburgh et al. [28] show that, in France, the pandemic has hit most hardly the most unequal communities.

3 States in the USA have implemented a variety of supply-side interventions to address the crisis, such as prescription drug monitoring programs, pain management clinics laws and naloxone access laws. In France, the Ministry of Health has forbidden OTC sales of codeine in 2017 and shortened the duration of tramadol prescriptions in 2020. Natali [46] evaluates the impact of restricting access to OTC codeine products and shows how the magnitude of responses to this policy varies depending on the prevalence of poverty at the local level.

4 *OpenHealth* is a company with headquarters in France that collects health data and make them available for health authorities, researchers and the general public.

5 Throughout the paper, we use the terms ‘opioid sales’, ‘opioid use’ and ‘opioid consumption’ interchangeably, even though we do not observe actual consumption by individual patients. This is a limitation of the present study.

6 Throughout the paper, we use the terms ‘active ingredient’ and ‘active substance’ interchangeably.

7 Areas with low doctors’ density are known in France as ‘medical deserts’.

8 Doctor shopping is defined as the practice of visiting multiple physicians to illicitly obtain multiple prescriptions. Likewise, pharmacy shopping is defined as the practice of visiting multiple pharmacies to obtain more medications.

9 However, none of the products under analysis in this paper can be advertised through medias since the French law forbids the advertisement of narcotic drugs to the general public.

10 At the European level, the regulatory agency in charge of providing marketing authorization is the European Medicines Agency (EMA). At the national level, this is the National Agency for the Safety of Medicines and Health Products (Agence Nationale de Sécurité du Médicament et des Produits de Santé, ANSM).

11 Article L. 5121-8 et L. 5121-9 du code de la santé publique.

12 Article R. 163-5 I 2 du code de la sécurité sociale.

13 Article R. 163-16-4 du code de la sécurité sociale.

14 Article R. 163-2 du code de la sécurité sociale.

15 Notice that these instruments have been substituted by ‘Complémentaire Santé Solidaire (CSS)’ in 2019.

16 The *OpenHealth* database provides data on sales by community pharmacies only (retail sales), thus neglecting hospital usage. In addition, Corsica and overseas departments are not included in our database. Note also that ‘departments’ is the denomination France uses for districts.

17 If the DDD for a particular active ingredient was not mentioned on this website (this happens most frequently for codeine combinations), we contacted the WHO directly and applied the DDD that they suggested.

18 For France, this is the median income in Metropolitan France.

19 Tables 8 and 9 in the appendix provide the list of companies for each active ingredient. Since some products were introduced after 2008 or withdrawn before 2017, the number of observations in Table 1 is sometimes smaller than the number of companies, multiplied by 94 departments and 10 years. For example, during our study period, there were four companies active on the oxycodone market. However, only Mundipharma was active during the whole study period, while the competitors entered on the market later, yielding a total number of observations equal to 1692.

20 Notice that Mundipharma is the European denomination for Purdue Pharma, the firm marketing Oxycontin.

21 Even though codeine and tramadol are considered mild opioids, the addiction risks linked to their use remain serious.

22 Tables 7, 8, 9 in Appendix 1 list the names of the active ingredients and product denominations, as well as all the companies active in the country. We include the following active substances in our analysis: tramadol (alone or in combination), codeine combinations, oxycodone, fentanyl and morphine. Results for transdermal and transmucosal fentanyl as well as for oral and injectable morphine are discussed separately. This is because different routes of administration require different DDDs for these ingredients.

23 This observation is consistent with the (somewhat counterintuitive) findings in Castanheira et al. [11] that when a molecule experiences generic entry, its overall consumption (originator + generics) decreases because part of the demand switches to the generic version of the substance, while part of the patients switches to its closest substitute (fentanyl, in this case). The authors rationalize this pattern by a decrease in promotion expenditures by the incumbent when its patent is close to expiration.

24 Notice that, for some products in some departments and time periods, sales are equal to zero. For this reason, we consider the logarithm of DDDs consumed per 1000 inhabitants plus one. Moreover, some products were introduced on the French market later than 2008 or withdrawn before 2017. These observations are treated as missing in our dataset. This allows us to distinguish between observations that are missing because the product was not on the market and observations for which sales were equal to zero.

25 In France, direct-to-physician advertising is allowed, while direct-to-consumer advertising is forbidden.

26 The company-by-year fixed effects, for example, would control for the average price of drugs by company *c*. As mentioned in “Institutional background”, however, the reimbursement rate for most products in our database is 65%. The remaining part can be paid through complementary insurance, which is owned by around 90% of French residents. Moreover, individuals on low income are entitled to receive free supplementary insurance.

27 This holds true for both mild and strong opioids, as well as for regressions considering each active ingredient separately. Tables 18 (mild opioids) and 19 (strong opioids) in Appendix 4 provide results for regressions that substitute the unemployment rate with its logarithmic transformation. Further results are available from the authors upon request.

28 Although our data suggest a linear relationship between (log) opioid sales and GPs density, we also tried regressions considering a quadratic relationship between the two. Results are showed in Tables 20 and 21 of Appendix 4.

29 Results are available from the authors upon request.

30 The interested reader can refer to Santos Silva and Tenreyro [57] for a comparison between PPML and log-linear models.

31 For further insights on opioid-related deaths by age group in France, see Appendix 2.

32 The number of opioid-related deaths per million people is equal to 91.1 in the USA in 2017 (22.4 in 1999), which is 23 times larger than the 3.8 in France in 2016 (1.3 in 2000) (see “Opioid-related death”).
